# Identification of cross‐reactive antibodies for the detection of lymphocytes, myeloid cells and haematopoietic precursors in the naked mole rat

**DOI:** 10.1002/eji.201948124

**Published:** 2019-09-05

**Authors:** Yury Shebzukhov, Susanne Holtze, Heike Hirseland, Hubert Schäfer, Andreas Radbruch, Thomas Hildebrandt, Andreas Grützkau

**Affiliations:** ^1^ Deutsches Rheuma‐Forschungszentrum Berlin (DRFZ) ein Institut der Leibniz‐Gemeinschaft Berlin Germany; ^2^ Department of Immunology Lomonosov Moscow State University (MSU) Moscow Russia; ^3^ Leibniz‐Institut für Zoo‐und Wildtierforschung (IZW) Berlin Germany; ^4^ Robert Koch‐Institut (RKI) Berlin Germany

**Keywords:** granulocytes, haematopoietic precursors, lymphocytes, myeloid cells, naked mole rat

## Abstract

The naked mole rat (*Heterocephalus glaber*, NMR) is a rodent with exceptional longevity, low rates of age‐related diseases and spontaneous carcinogenesis. The NMR represents an attractive animal model in longevity and cancer research, but there are no NMR‐specific antibodies available to study its immune system with respect to age‐ and cancer‐related questions. Substantial homology of major NMR immune cell markers with those of Guinea pig, human and, to a lesser extent, mouse and rat origin are implicated for the existence of immunological cross‐reactivity. We identified 10 antibodies recognising eight immunophenotypic markers expressed on the NMR's T and B lymphocytes, macrophages/monocytes and putative haematopoietic precursors and used them for an immunophenotyping of leukocyte subsets of peripheral blood, spleen and bone marrow samples. Overall, we found that the leukocyte composition of NMR peripheral blood is comparable to that of mice. Notably, the frequency of cytotoxic T cells was found to be lower in the NMR compared to corresponding mouse tissues and human blood. Antibodies used in the present paper are available either commercially or from the scientific community and will provide new opportunities for the NMR as a model system in ageing‐ and cancer‐related research areas.

## Graphical abstract

We identified cross‐reactive antibodies, recognising immune cell‐related marker molecules that allow the investigation of lymphocytes, monocytes/macrophages and haematopoietic cells in blood and lymphoid organs of the long‐lived naked mole rat (NMR). These antibodies are basic research tools to use the NMR as promising animal model to study ageing‐ and cancer‐related research questions.

(Photo of NMR by Roland Gockel)

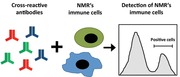

## Introduction

The naked mole rat (NMR) is a novel rodent model to study longevity and resistance to cancer. The NMR's lifespan is about 30 years, which is five times longer than expected based on its body size, and exhibits neither significant senescence nor an age‐related increase in mortality 1. Moreover, spontaneous carcinogenesis was observed only recently in aged animals 2, 3. Several intrinsic protective cancer‐related molecular mechanisms of the NMR were reported, including activity of the INK4 locus and mutation of the ERAS oncogene 4, 5, but they were not related to the NMR immune system.

Age‐related changes of the immune system in humans and mice are mainly caused by reduced thymus activity and a general increase of inflammaging – a chronic low‐grade inflammation caused by up‐regulated activity of the innate immune system 6, 7. It was shown recently that, in contrast to humans and mice, T‐cell receptor diversity does not decrease with age in another long‐living rodent, *Spalax*. 8.

To date, it has not been possible to investigate the role of the NMR immune system in resistance to ageing and cancer, since no specific reagents, particularly NMR‐specific antibodies, are available. In this study, we screened numerous antibody clones for cross‐reactivity with NMR leukocyte antigens, which allowed us for the first time to perform basic immunophenotyping of NMR leukocytes with respect to T and B cells, macrophages and putative haematopoietic precursors in different immune‐relevant tissues.

## Results and discussion

### Identification of antibodies recognising NMR immune cells markers

At first, we analysed immune cell‐related NMR, Guinea pig, human, mouse and rat surface antigens to identify those with high and intermediate homology of extracellular domains (see Supporting Information Table 1). For 18 selected markers, we cloned target NMR proteins and screened antibodies for their cross‐reactivity with NMR antigens using transfected cell lines (Screening strategy I, Supporting Information Tables 1 and 2). Mouse monoclonal antibodies against human CD14 and CD34 and guinea pig CD8α recognised mouse T cells (RLM‐11 T‐cell line) expressing corresponding NMR antigens (Table 1, examples are shown in Supporting Information Fig. 1A–C) and populations of primary NMR immune cells (examples are shown in Supporting Information Fig. 1D).

**Table 1:** Antibodies recognising NMR immune cell markers

Antibody/source	Clone/isotype conjugation	Working concentration	Identified cell type	Confirmed by
Rat anti‐human CD3ε	CD3‐12, IgG1 unconjugated	10 μg/mL	T cells, staining of intracellular domain	Recognition of transfected HEK293T cells and primary NMR cells
Mouse anti‐human CD3ε, RKI*	PC3/188a, IgG1, unconjugated^#^	20 μg/mL	T cells, staining of intracellular domain	Recognition of transfected HEK293T cells
Mouse anti‐Guinea pig CD8α	CT6, IgG1 unconjugated	10 μg/mL	Cytotoxic T cells	Recognition of transfected RLM11 cells and primary NMR cells
Mouse anti‐human CD14, DRFZ	TM1, IgG1, Cy5	2.5 μg/mL	Monocytes, macrophages	Recognition of transfected RLM11 cells and primary NMR cells
Mouse anti‐human CD34,	AC136, IgG2a APC	Diluted 1:20**	Haematopoietic cells (including human HSC), non‐haematopoietic progenitor and stem cells	Recognition of transfected RLM11 cells and primary NMR cells
Rat anti‐mouse CD117/c‐Kit, DRFZ	ACK4, IgG2a Cy5^#^ and PE‐Cy5	20 μg/mL	HSC, multipotent progenitors (MPP) and common myeloid progenitors (CMP)	Q‐RT‐PCR on sorted NMR cells
Mouse anti‐GP IgM, RKI*	31D2, IgG PE or unconjugated^#^	2.5 μg/mL	B cells	Q‐RT‐PCR on sorted NMR cells
Mouse anti‐GP MHC‐II, RKI*	27E7 unconjugated^#^	5–10 μg/mL	Macrophages, B cells	Q‐RT‐PCR on sorted NMR cells
Mouse anti‐GP MHC‐II, RKI*	MSgp8 Pacific Blue or unconjugated^#^	5–10 μg/mL	Macrophages, B cells	Q‐RT‐PCR on sorted NMR cells
Hamster anti‐mouse CXCR3	CXCR3‐173, IgG PE	2 μg/mL	Th1‐type CD4^+^ T cells and effector CD8α^+^ T cells	Q‐RT‐PCR on sorted NMR cells

* Provided by Dr. Hubert Schäfer (RKI, Berlin).

** Miltenyi Biotec cat. # 130‐113‐738.

# Supporting Information only.

The extracellular domain of the NMR CD3ε chain has low homology with other tested species (Supporting Information Table 1), but its intracellular part contains an evolutionary conserved C‐terminal epitope recognised by the clones CD3‐12 (rat anti‐human CD3ε) and PC3/188a (mouse anti‐human CD3ε) (Supporting Information Fig. 2A, Table 1). The clone CD3‐12 showed a stronger staining of human HEK293T cells expressing the NMR CD3ε (Supporting Information Fig. 2A) and was selected for further analysis (Supporting Information Fig. 2B).

We then screened the antibodies for their reactivity towards primary NMR immune cells (Screening strategy II). To verify specificity of antibody staining, positively and negatively stained cell populations were isolated by fluorescence‐activated cell sorting and analysed by Q‐RT‐PCR for the expression of the surface molecule of interest (an example is shown in Supporting Information Fig. 3). Through this approach, we were able to identify five additional antibody clones recognising surface IgM, MHC class II (MHC‐II) antigens (two clones), the chemokine receptor CXCR3 and the proto‐oncogene c‐Kit/CD117 (Table 1). Finally, specificity of these five antibodies was confirmed by sequencing the appropriate Q‐RT‐PCR products. In total, we screened 96 monoclonal antibodies against 38 Guinea pig, human and mouse immune cell markers (Supporting Information Table 2) and came up with 10 antibodies, recognising eight NMR antigens (Table 1).

### Analysis of NMR leukocytes

**Figure 1:**
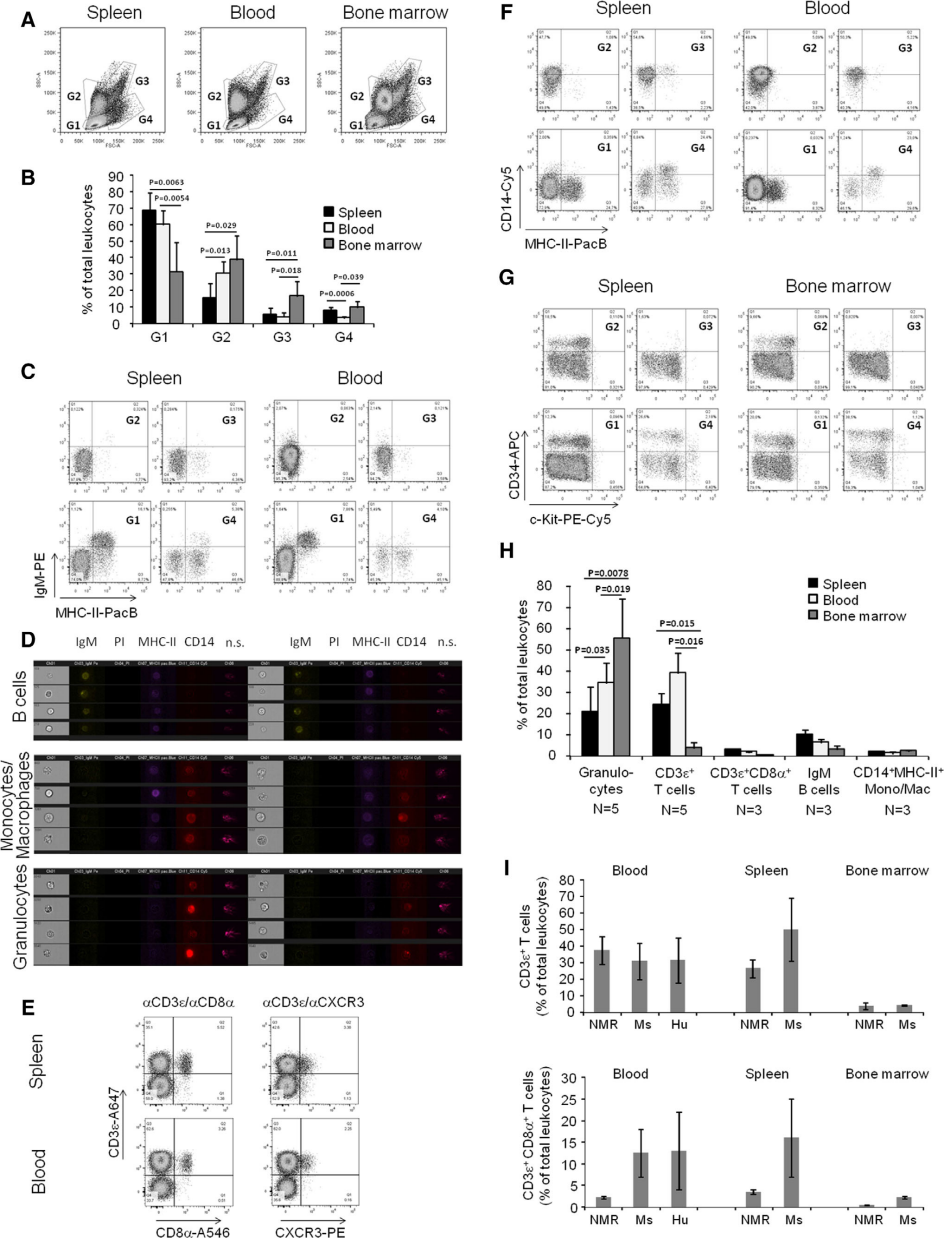
Flow cytometry analysis of NMR leukocytes. Cells were isolated from the NMR spleen, blood and bone marrow and stained as described in Materials and Methods and in Table 1. Cells were analysed by flow cytometry for: (A, B) distribution of size (forward scatter/FSC‐A) and granularity (side scatter/SSC‐A) by conventional flow cytometry. A total of five NMR samples were analysed in four independent experiments (A). Representative results of one of four experiments. (B) Data of four experiments with five NMR samples. (C) Expression of IgM and MHC‐II in G1‐G4 sub‐populations. (D) Size, granularity and expression of IgM, MHC‐II and CD14 analysed by imaging flow cytometry. Representative results of one of two experiments with a total of two NMR samples are shown. n.s. – non‐specific binding. Images also including cells from NMR blood and spleen are shown in Supporting Information Fig. S6. (E) Expression of CD3ε, CD8α and CXCR3 in G1 sub‐population. (F) Expression of CD14 and MHC‐II in G1‐G4 sub‐populations. (G) Expression of CD34 and c‐Kit/CD117 in G1‐G4 sub‐populations. (C, E‐G) Representative results of one of three experiments with a total of three NMR samples are shown. Additional samples from bone marrow (C, E, F) and blood (G) and corresponding unstained controls are shown in Supporting Information Figs. 5 (C), S8 (E), S9 (F) and S10 (G). (H) Percentages of granulocytes, CD3ε^+^ T cells, CD3ε^+^CD8α^+^ T cells, IgM^+^ B cells and CD14^+^MHC‐II^+^ monocytes/macrophages are shown. Data from three independent experiments are shown. N – number of analysed NMR samples. (B, H) Data are shown as mean ± standard deviation (SD). Student's *t*‐test was used for statistical analysis. The difference is considered statistically significant if *p* < 0.05. (I) Frequency of total CD3ε^+^ (upper panel) and cytotoxic CD3ε^+^CD8α^+^ (lower panel) T cells in NMR, mouse (Ms) and human (Hu) organs. Data from three independent experiments are shown for NMR (five and three samples were analysed accordingly for total CD3ε^+^ and cytotoxic CD3ε^+^CD8α^+^ T cells). Data for the mouse (averaged for CB17, C57BL/6 and BALB/c strains) and humans are taken from the literature sources^19^, ^20^, ^21^, ^22^. Data are shown as mean ± SD. Gating strategy is shown in Supporting Information Figs. 4 (C, E, F and G) and S6 (D).

After exclusion of debris, non‐lysed erythrocytes, aggregated and dead cells, we identified four sub‐populations of NMR immune cells according to their forward (FSC‐A) and side (SSC‐A) scatter characteristics, reflecting differences in cell size and granularity: FSC‐A‐low/SSC‐A‐low (**G1**), FSC‐A‐low/SSC‐A‐high (**G2**), FSC‐A‐high/SSC‐A‐high (**G3**) and FSC‐A‐high/SSC‐A‐int. (intermediate) (**G4**) (Fig. 1A and B).

#### B and T lymphocytes

To identify B lymphocytes, we stained primary NMR immune cells with antibodies recognising surface IgM (clone 31D2) and MHC‐II (clone MSgp8). The majority of IgM^+^ cells were found in the **G1** sub‐population. The highest proportion was found in spleen, followed by blood and bone marrow. Almost all IgM^+^ cells co‐expressed the MHC‐II antigen (Fig. 1C, Supporting Information Fig. 5).

Analysis of double‐positive IgM^+^/MHC‐II^+^ cells by imaging flow cytometry confirmed their relative small size and low granularity (Fig. 1D, Supporting Information Fig. 7A). We concluded that IgM^+^ MHC‐II^+^ cells represent at least a subpopulation of NMR B lymphocytes.

For the identification of NMR T lymphocytes, we used an unconjugated αCD3ε rat primary antibody (clone CD3‐12) and an α‐rat IgG A647 secondary antibody. Cells were pre‐stained with antibodies recognising CD8α (clone CT6) or CXCR3 (clone CXCR3‐173). Since CD3ε^+^ cells are of low granularity (Supporting Information Fig. 2B), only the **G1** sub‐population was analysed.

The highest relative number of CD3ε^+^ cells in the **G1** sub‐population was found in blood, followed by spleen and bone marrow, whilst the highest frequency of CD3ε^+^CD8α^+^ cells was observed in spleen, followed by blood and bone marrow (Fig. 1E, Supporting Information Fig. 8). Notably, the relative number of CD3ε^+^CD8α^+^ cells was surprisingly low in all tested NMR tissues (Fig. 1E, Supporting Information Fig. 8), and the nature of CD3ε^+^CD8α^−^ cells still has to be identified. Since CD3ε and CD8α can also be expressed by other cells such as γδT, natural killers (NK), dendritic (DC) and innate lymphoid (ILC) cells, additional markers are required for a fine analysis of CD3‐expressing cell subsets. However, it was recently shown that the NK cells and NK‐related MHC‐I receptors are absent in the NMR 9.

Co‐staining with αCXCR3 antibodies identified CD3ε^+^CXCR3^+^ cells representing putative effector cytotoxic T cells and/or Th1 lymphocytes (Fig. 1E, Supporting Information Fig. 8) 10. Further molecular analysis, for example for the expression of IFN‐γ or the Th1 master transcription factor T‐bet, is necessary to clarify our assumption, however.

#### Monocytes/macrophages

For the identification of monocytes/macrophages, primary NMR immune cells were stained with antibodies recognising MHC‐II and CD14 (clone TM1). CD14^+^ cells were found mainly in the **G2**, **G3** and **G4** sub‐populations (Fig. 1F, Supporting Information Fig. 9). Surprisingly, staining with αCD14 antibodies induced a shift of the entire **G2** and **G3** sub‐populations, presumably representing granulocytes. A significant proportion of cells in these sub‐populations appeared to be CD14^+^ in accordance with data from single‐cell gene expression profiling 9, but only a few cells expressed MHC‐II. By contrast, the **G4** sub‐population most probably represents monocytes and macrophages characterised by the co‐expression of CD14^+^ cells and the MHC‐II antigen (Fig. 1F, Supporting Information Fig. 9).

Morphological analysis of cells by imaging flow cytometry confirmed that CD14^+^MHC‐II^+^ double‐positive cells are larger and therefore very likely represent monocytes/macrophages, whereas CD14^+^MHC‐II^−^ cells showed higher granularity and most probably represent granulocytes (Fig. 1D, Supporting Information Fig. 7B and C).

In mouse and human granulocytes, the expression of CD14 is inducible under pathological conditions such as myelodysplastic syndrome [11]. The constitutive expression of CD14 on NMR granulocytes is possibly linked to a lower bacterial load of gut microbiota compared to mice and humans 12.

#### Putative haematopoietic stem cells

For the identification of putative NMR haematopoietic stem cells (HSC), we used antibodies recognising major markers of human (CD34) and mouse (c‐Kit) HSC 13, 14. Staining for CD34 and c‐Kit identified three different cell subsets in spleen and bone marrow and two subsets in blood (Fig. 1G, Supporting Information Fig. 12). CD34^+^ cells were mainly negative for c‐Kit and appeared in spleen and bone marrow as a separate subset in all sub‐populations of G1, G2 and G4. The highest number of CD34^+^ cells and the highest expression level of CD34 were found in G4 cells of spleen and bone marrow (Fig. 1G, Supporting Information Fig. 10A).

Defined subsets of c‐Kit^+^ cells were only found in **G4** cells of spleen (8.5 ± 0.1 %) and bone marrow (1.6 ± 0.7 %); these cells were negative or had an intermediate expression level of CD34, and double‐positive CD34^+^c‐Kit^+^ cells were not recognised as a distinct population (Fig. 1G, Supporting Information Fig. 10A).

In blood, some CD34^+^ and c‐Kit^+^ cells were also observed, but they did not form clearly defined subsets, and their CD34 and c‐Kit expression levels were lower compared to cells in corresponding spleen and bone marrow samples (Supporting Information Fig. 10A).

### Leukocyte counts in NMR spleen, blood and bone marrow

Finally, we used the NMR‐cross‐reactive antibodies identified so far to estimate the leukocyte composition of bone marrow, spleen and peripheral blood. No cell‐specific markers for granulocytes could be identified, unfortunately, meaning that they were only detectable according to their characteristic side‐scatter properties. In bone marrow, granulocytes represented the majority of leukocytes, whilst lymphocytes (G1 population) were in the majority in blood and spleen (Fig. 1B–H).

In spleen and blood, CD3ε^+^ T cells showed frequencies comparable to SSC^high^ granulocytes, followed by IgM^+^MHC‐II^+^ B cells (Fig. 1H). We found comparable numbers of T and B lymphocytes in bone marrow. Compared to mouse organs and human blood, only low frequencies of cytotoxic CD8α^+^ T cells and MHC‐II^+^CD14^+^ monocytes/macrophages were detectable in NMR blood and tissue samples (Fig. 1H and I, Table 2).

**Table 2:** Features of the NMR immune system compared to the mouse and humans

Organ/cell type/ functional parameter	NMR compared to mouse and human	References (*NMR related)
Blood	Lymphocytes represent a dominant leukocyte subset similar to mouse, in contrast to human blood. Lower frequency of monocytes and macrophages compared to mouse and human blood.	23 19, 20, 22
Spleen	Lower frequency of lymphocytes and higher frequency of granulocytes compared to mouse spleen. High expression of CD14 on myeloid cells.	19, 20 9*
Bone marrow	Similar proportions between granulocytes and lymphocytes compared to mouse.	22
Lymphocytes	Lower frequency of CD8α^+^ cytotoxic T cells compared to corresponding mouse tissues and human blood. Absence of NK cells and NK‐related MHC‐I receptors.	19, 20, 21, 22 9*
Macrophages	Higher functional activity of NMR bone marrow‐derived macrophages compared to mouse samples.	15*
Granulocytes	Expression of CD14 on NMR granulocytes, in contrast to healthy mice and humans.	11
Control of bacteria	Lower bacterial load of gut microbiota (102 ‐ 105 CFU/g) compared to mice (106 ‐ 109 CFU/g) and humans (108 ‐ 1012 CFU/g). Lower inflammogenic potential of NMR gut microbiota compared to mouse and human samples.	12*
Control of viruses	Susceptibility to coronavirus and to Herpes simplex virus type 1 (HSV1).	24, 25*
Control of parasites	Susceptibility to intracellular parasite *Leishmania donovani* similar to humans and some mouse strains.	26*

Results of the comparative analysis of leukocyte composition of NMR and mouse tissues and human blood and known functional features of NMR immune system are summarised in Table 2. These data, along with data from Cheng *et al*.^15^ and Hilton *et al*. 9, indicate that the innate arm of the NMR immune system plays a more dominant role in the host defence as compared to that of the mouse and human.

### Concluding remarks

This study presents for the first time an immunophenotyping of NMR leukocytes in different immune‐related tissues with a set of cross‐reactive antibodies. Using these antibodies together with SSC and FSC scatter characteristics, we were able to recognise around 80, 65 and 60% of blood, bone marrow and spleen leukocytes respectively. We are aware that additional antibodies are necessary for an in‐depth analysis of the entire cellular immune system of the NMR, including those reflecting differentiation‐ and activation‐dependent phenotypes. In particular, antibodies recognising extracellular domains of CD3ε are needed because expression of intracellular CD3ε does not allow discrimination between membrane‐ and cytoplasm‐associated isoforms as observed in immature T‐cell subsets 16. Finally, a functional characterisation of identified NMR cell types will be necessary to reveal differences and similarities to those in mice and humans.

## Materials and methods

### Animals

NMR colonies are maintained at the Leibniz‐Institut für Zoo‐und Wildtierforschung (IZW), Berlin, Germany, in heated (26–29°C) acrylic glass artificial burrow systems with tunnels and chambers at a relative humidity of 60–80 %. The chamber bedding consists of wood shavings, twigs and unbleached paper tissue. Fresh food including sweet potatoes, carrots, fennel, apples, oat flakes and cereal supplement containing vitamins and minerals is given daily ad libitum. NMR euthanasia and tissue sampling were approved by the local Ethics Committee of the Regional Office for Health and Social Affairs Berlin, Germany, (#ZH 156). Overall, 15 animals of both genders at ages ranging from six months to five years were euthanised and used for experiments. All animals were from different litters.

### Cloning of NMR cell markers

Total RNA was isolated from snap‐frozen spleen samples using Tri‐Reagent (Sigma‐Aldrich, St. Louis, MO), treated with RNase‐free DNase I (Thermo Scientific, Waltham, MA) and converted to cDNA using RevertAid H Minus Reverse Transcriptase (Thermo Scientific) and random nonamer primers (Life Technologies, Grand Island, NY). Coding sequences were amplified using Phusion High‐Fidelity DNA Polymerase (Thermo Scientific) and gene‐specific primers with additional sequence encoding FLAG‐tag (Asp‐Tyr‐Lys‐Asp‐Asp‐Asp‐Asp‐Lys) (Supporting Information Table S2) and cloned to pMSCV‐IRES‐GFP vector (https://www.addgene.org/20672/).

### Transfection of cell lines

Mouse T‐cell line RLM11 was transfected using Amaxa Nucleofector and Amaxa Cell Line Kit L (Lonza, Basel, Switzerland) according to the manufacturer's instructions. Human kidney embryonic fibroblasts HEK293T were transfected using the Ca^2+^‐phosphate method 17.

### Conventional and image flow cytometry analysis and isolation of NMR cell populations

Total cells isolated from spleen, blood and bone marrow were incubated on ice in RBC Lysis Buffer (155 mM NH_4_Cl, 10 mM KHCO_3_, 0.1 mM EDTA, pH 7.3) for 10–15 min (until the lysis of erythrocytes). Leukocytes were washed by PBS containing 0.1 % BSA and 2 mM EDTA and incubated with antibodies listed in Table 1 and Supporting Information Table 3. Goat anti‐rat IgG Alexa Fluor 647‐conjugated secondary antibodies (final concentration 2 μg/mL) (Jackson ImmunoResearch, West Grove, PA) with minimal cross‐reaction with human, bovine, horse, mouse and rabbit serum proteins (α‐rat IgG A647) and goat anti‐mouse IgG Alexa Fluor 546‐conjugated secondary antibodies (final concentration 10 μg/mL) (Thermo Scientific) with minimal cross‐reaction with human IgG and serum proteins (α‐mouse IgG A546) were used with corresponding unconjugated primary antibodies. To avoid unspecific binding of antibodies, cells were pre‐treated with 10x excess of total control IgG antibodies of the same origin as the primary antibody (for direct staining) or as the secondary antibody (for indirect staining). To exclude dead cells, propidium iodide was used for unfixed cells and Fixable Viability Dye eFluor^®^ 780 (eBioscience, San Diego, CA) for fixed cells. The Fixation & Permeabilization Buffer Set (eBioscience, San Diego, CA) was used according to the manufacturer's instructions for intracellular staining.

Stained cells were analysed by a FACSCanto cytometer (BD, Franklin Lakes, NJ), an ImageStream^®^X imaging flow cytometer (Amnis/EMD Millipore, Seattle, WA) or isolated by a FACSAria II sorter (BD). Antibodies are listed in Table 1 and Supporting Information Table 3. Antibodies from DRFZ and RKI depositories were conjugated in house as mentioned in Table 1 and Supporting Information Table 3. Gating strategy for conventional flow cytometry is shown in the Supporting Information Fig. 4, and for image flow cytometry in the Supporting Information Fig. 6. Results of conventional and image flow cytometry were analysed accordingly with FlowJo v10 (FlowJo LCC, Ashland, OR) and IDEAS^TM^ (Amnis/EMD Millipore) software. All experiments were performed according the Guidelines for the use of flow cytometry and cell sorting in immunological studies 18.

### Quantitative mRNA analysis

RNA was isolated from sorted cells and converted to cDNA as described above. Q‐PCR was performed in a StepOne Plus^TM^ (Applied Biosystems, Foster City, CA) Real‐Time PCR System using Maxima SYBR Green/ROX qPCR Master Mix (Thermo Scientific). Primers are listed in Supporting Information Table 2. All mRNA data were normalised to NMR β‐actin and GAPDH.

### DNA sequencing

Cloned NMR antigens and Q‐RT‐PCR products were sequenced by GATC Biotech AG (Konstanz, Germany).

### Statistical analysis

Statistical analysis was performed by Microsoft Excel software using paired Student's *t*‐test.

## Conflict of interest

The authors declare no financial or commercial conflict of interest.

**Abbreviations**
FACS: fluorescence‐activated cell sorting
HSC: haematopoietic stem cells
NMR: naked mole rat
Q‐RT‐PCR: reverse transcription and quantitative polymerase chain reaction

## Supporting information

Figure S1A. Antibodies recognising NMR surface antigens expressed in RLM11 mouse T cells
Figure S2. Antibodies recognising the intracellular domain of NMR CD3e A.
Figure S3. Antibodies recognising NMR MHC‐II (example of Screening strategy II).
Figure S4. Gating strategy for conventional flow cytometry.
Figure S5. Identification of putative B cells (complete version of Figure 1C)
Figure S6. Gating strategy for imaging flow cytometry.
Figure S7A. Imaging flow cytometry analysis of NMR immune cells. Putative B cells.
Figure S7B. Imaging flow cytometry analysis of NMR immune cells. Putative monocytes/macrophages.
Figure S7C. Imaging flow cytometry analysis of NMR immune cells. CD14+ subset of putative granulocytes.
Figure S8. Identification of putative T cells (complete version of Figure 1E).
Figure S9. Identification of putative monocytes/macrophages (complete version of Figure 1F).
Figure S10A. Analysis of putative haematological precursors (complete version of Figure 1G).
Figure S10B. Analysis of putative haematological precursors (individual stainings – control of channels compensation).
Table S1. Homology of NMR immune cells markers with Guinea pig, human, mouse and rat antigens
Table S2. Primers Primers for Q‐RT‐PCR
Table S3. Screened antibodies
Table S3 (cont.). Screened antibodies
