# Supplementary material for: Identification of cross‐reactive antibodies for the detection of lymphocytes, myeloid cells and haematopoietic precursors in the naked mole rat
Source: Eur J Immunol. 2019 Sep 5;49(11):2103–10. doi: 10.1002/eji.201948124 (PMC7163560; doi:10.1002/eji.201948124)
Supplement: Supplementary file 1 — Figure S1A. Antibodies recognising NMR surface antigens expressed in RLM11 mouse T cells Figure S2. Antibodies recognising the intracellular domain of NMR CD3e A. Figure S3. Antibodies recognising NMR MHC‐II (example of Screening strategy II). Figure S4. Gating strategy for conventional flow cytometry. Figure S5. Identification of putative B cells (complete version of Figure 1C) Figure S6. Gating strategy for imaging flow cytometry. Figure S7A. Imaging flow cytometry analysis of NMR immune cells. Putative B cells. Figure S7B. Imaging flow cytometry analysis of NMR immune cells. Putative monocytes/macrophages. Figure S7C. Imaging flow cytometry analysis of NMR immune cells. CD14+ subset of putative granulocytes. Figure S8. Identification of putative T cells (complete version of Figure 1E). Figure S9. Identification of putative monocytes/macrophages (complete version of Figure 1F). Figure S10A. Analysis of putative haematological precursors (complete version of Figure 1G). Figure S10B. Analysis of putative haematological precursors (individual stainings – control of channels compensation). Table S1. Homology of NMR immune cells markers with Guinea pig, human, mouse and rat antigens Table S2. Primers Primers for Q‐RT‐PCR Table S3. Screened antibodies Table S3 (cont.). Screened antibodies [file EJI-49-2103-s001.pdf]

European Journal of Immunology

Supporting Information
for
DOI 10.1002/eji.201948124

Yury Shebzukhov, Susanne Holtze, Heike Hirseland, Hubert Schäfer,
Andreas Radbruch, Thomas Hildebrandt and Andreas Grützkau

Identification of cross-reactive antibodies for the detection of lymphocytes, myeloid cells and haematopoietic precursors in the naked mole rat

© 2019 The Authors. *European Journal of Immunology* published by WILEY-VCH Verlag GmbH & Co. KGaA, Weinheim.

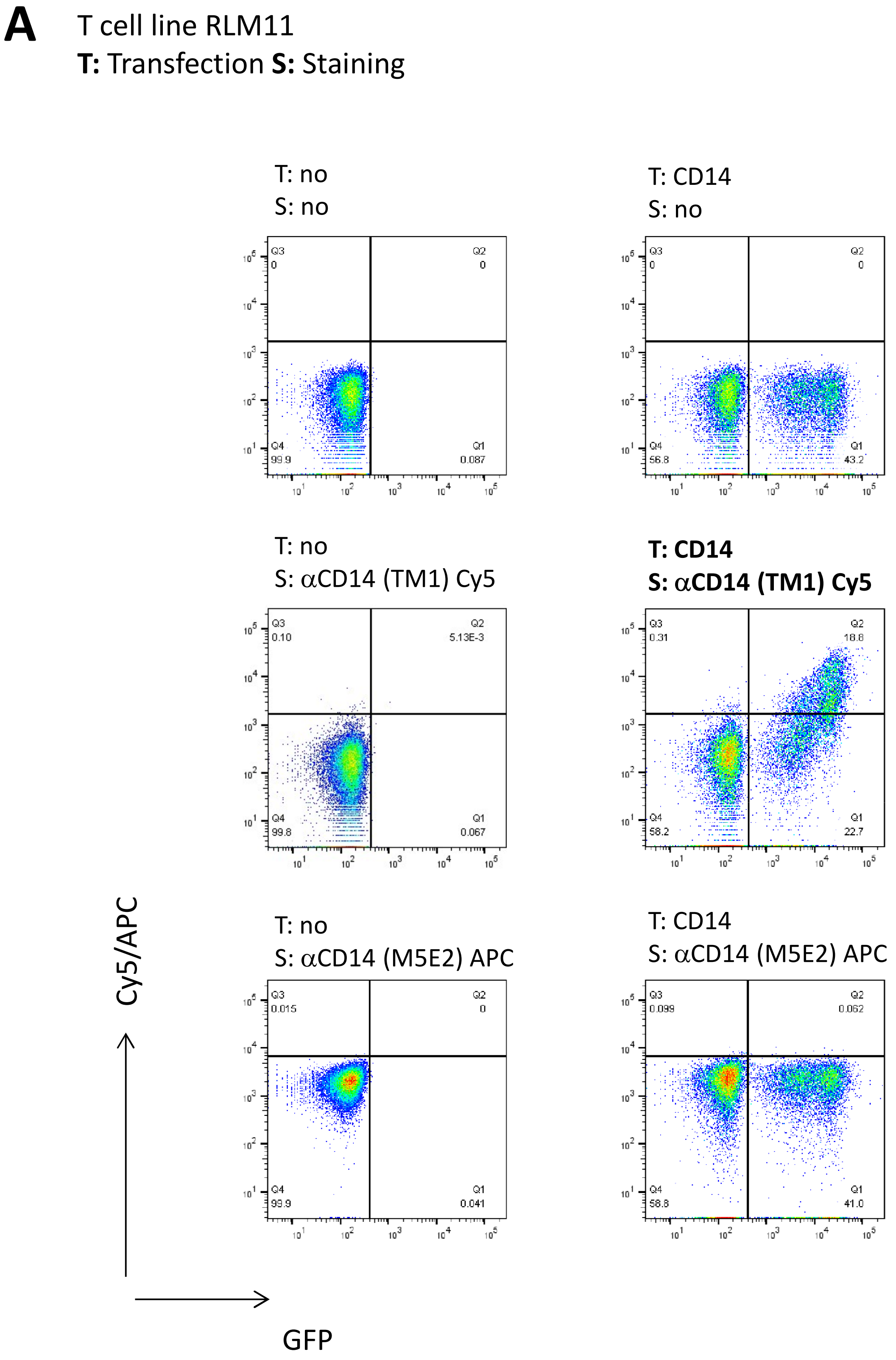

Supporting information Figure S1A. Antibodies recognising NMR surface antigens expressed in RLM11 mouse T cells

RLM11 mouse T cells were transfected with vector NMR CD14, stained with mouse antibodies recognising human CD14, clones TM1 and M5E2 (shown as negative control) and analysed by flow cytometry. Representative results from one of two experiments are shown. Gating strategy is shown in the Supporting Information Figure S4.

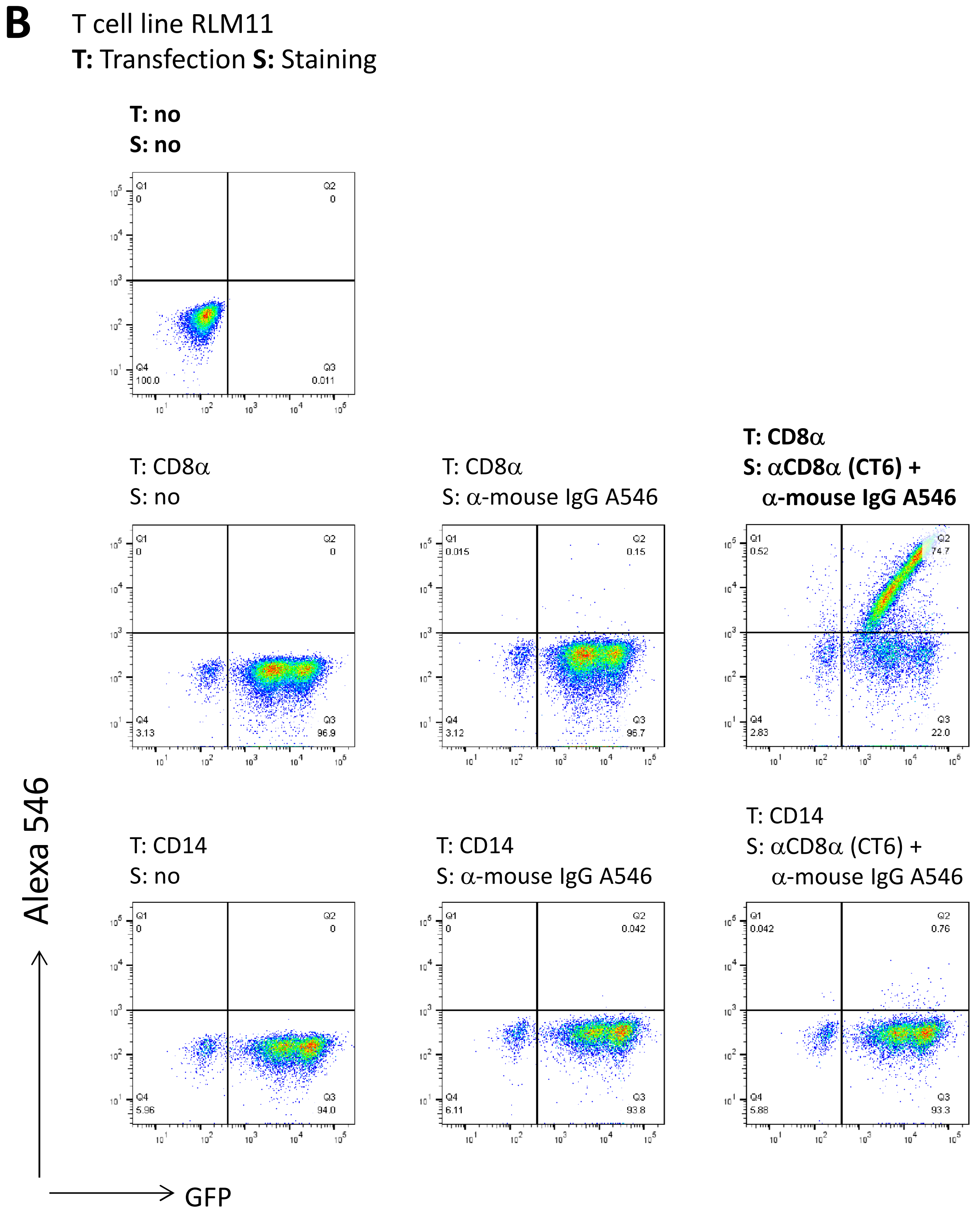

Supporting Information Figure S1B. Antibodies recognising NMR surface antigens expressed in RLM11 mouse T cells

RLM11 mouse T cells were transfected with vectors encoding NMR CD8α and CD14. Cells were stained with primary antibodies recognising Guinea pig CD8α (clone CT6) and α-mouse IgG A546 secondary antibodies and analysed by flow cytometry. Representative results from one of two experiments are shown. Gating strategy is shown in the Supporting Information Figure S4.

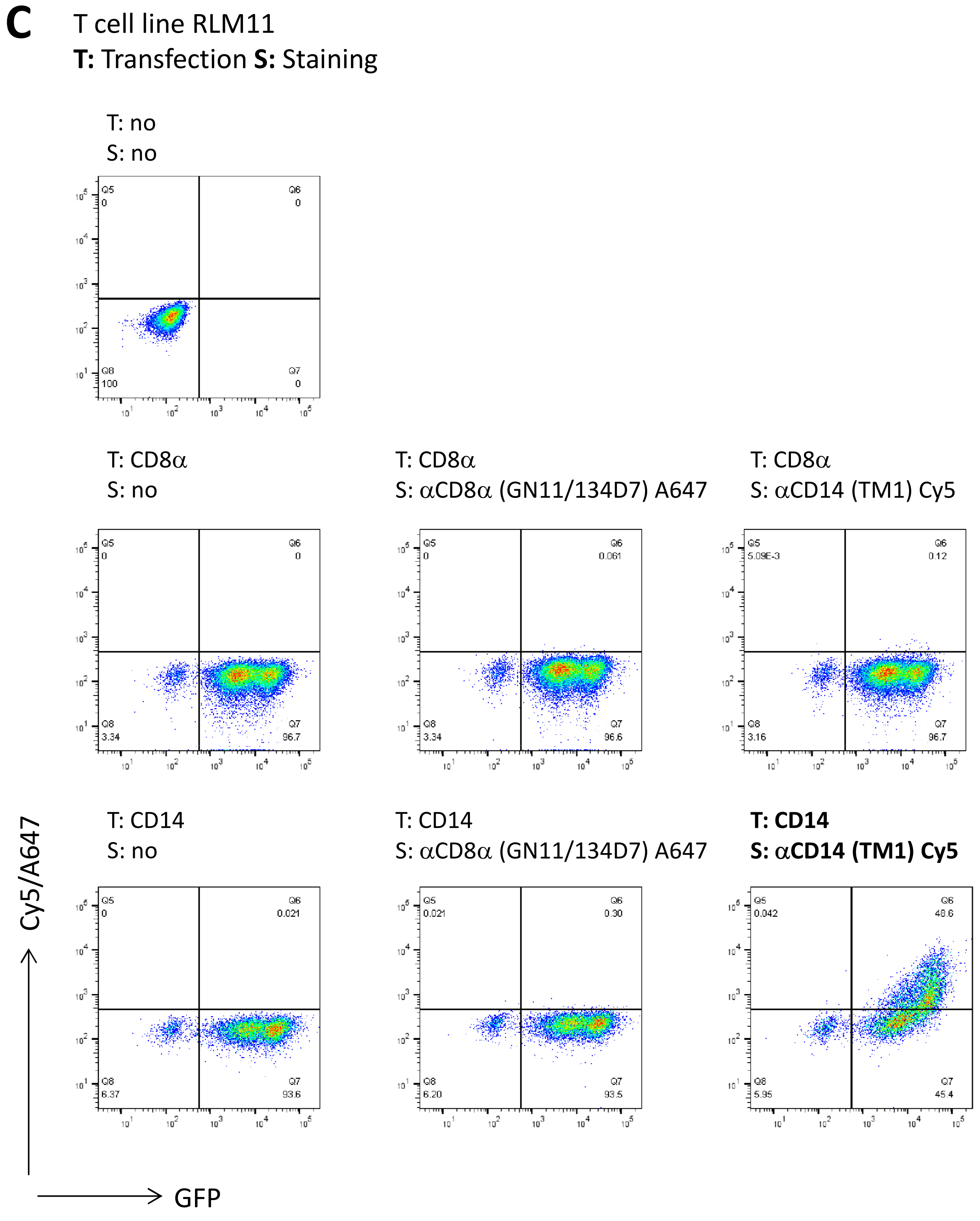

Supporting Information Figure S1C. Antibodies recognising NMR surface antigens expressed in RLM11 mouse T cells

RLM11 mouse T cells were transfected with vectors encoding NMR CD8α and CD14. Cells were stained with antibodies recognising human CD8α (clone GN11/134D7, shown as negative control) and CD14 (clone TM1) and analysed by flow cytometry. Representative results from one of two experiments are shown. Gating strategy is shown in the Supporting Information Figure S4.

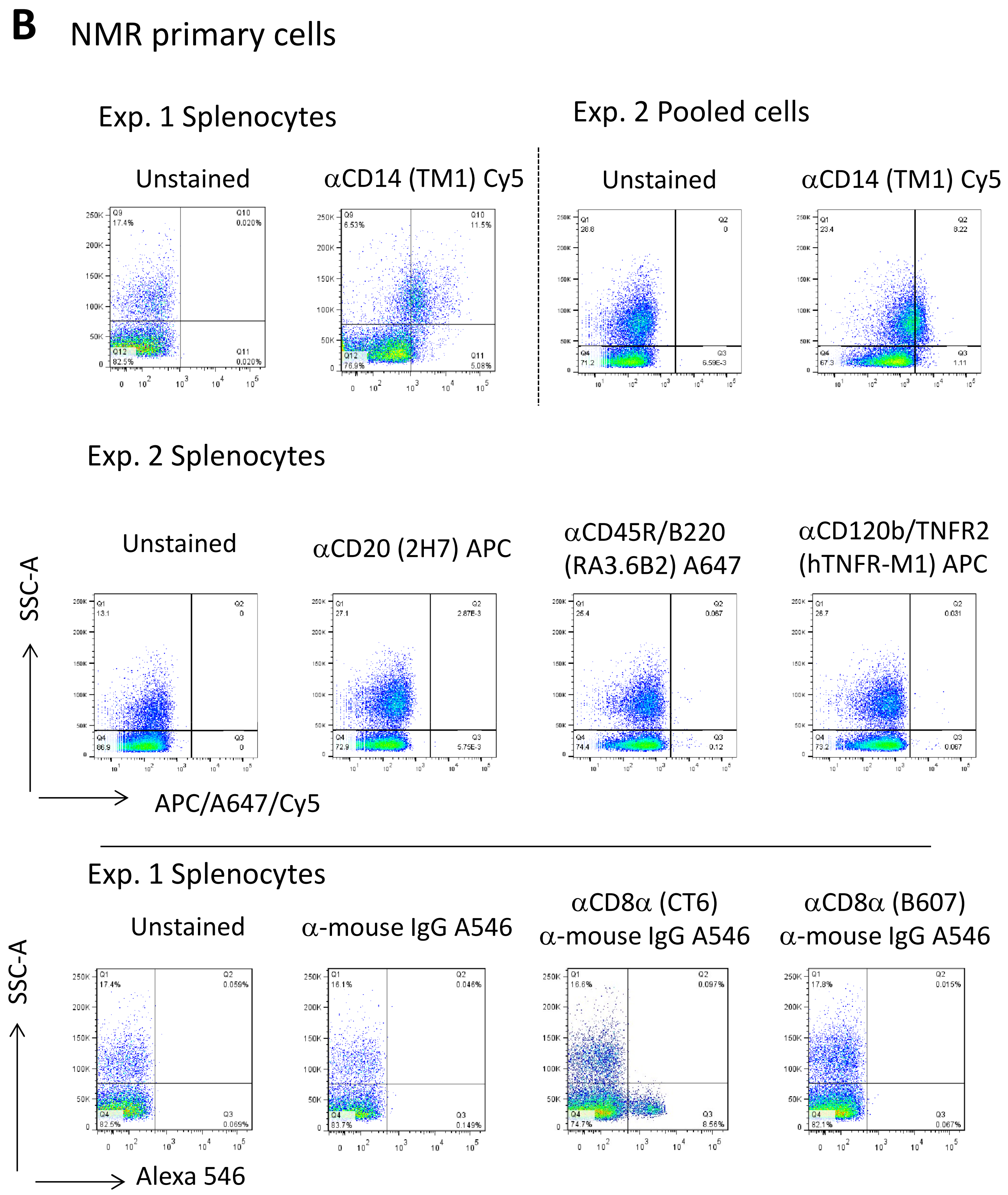

Supplementary Figure S1D Antibodies recognising primary NMR cells

Cells were isolated from NMR blood, spleen and bone marrow. Cells were stained with antibodies recognising Guinea pig CD8α, human CD14, CD20, CD45R/B220 and CD120b/TNFR2 and analysed by flow cytometry. Clone B607 recognising Guinea pig CD8α and antibodies, recognising human CD14, CD20, CD45R/B220 and CD120b/TNFR2 are shown as negative control. α-Mouse IgG A546 secondary antibodies were used with antibodies against CD8α. Representative results from one of two experiments with a total of two NMR samples are shown. Gating strategy is shown in the Supporting Information Figure S4.

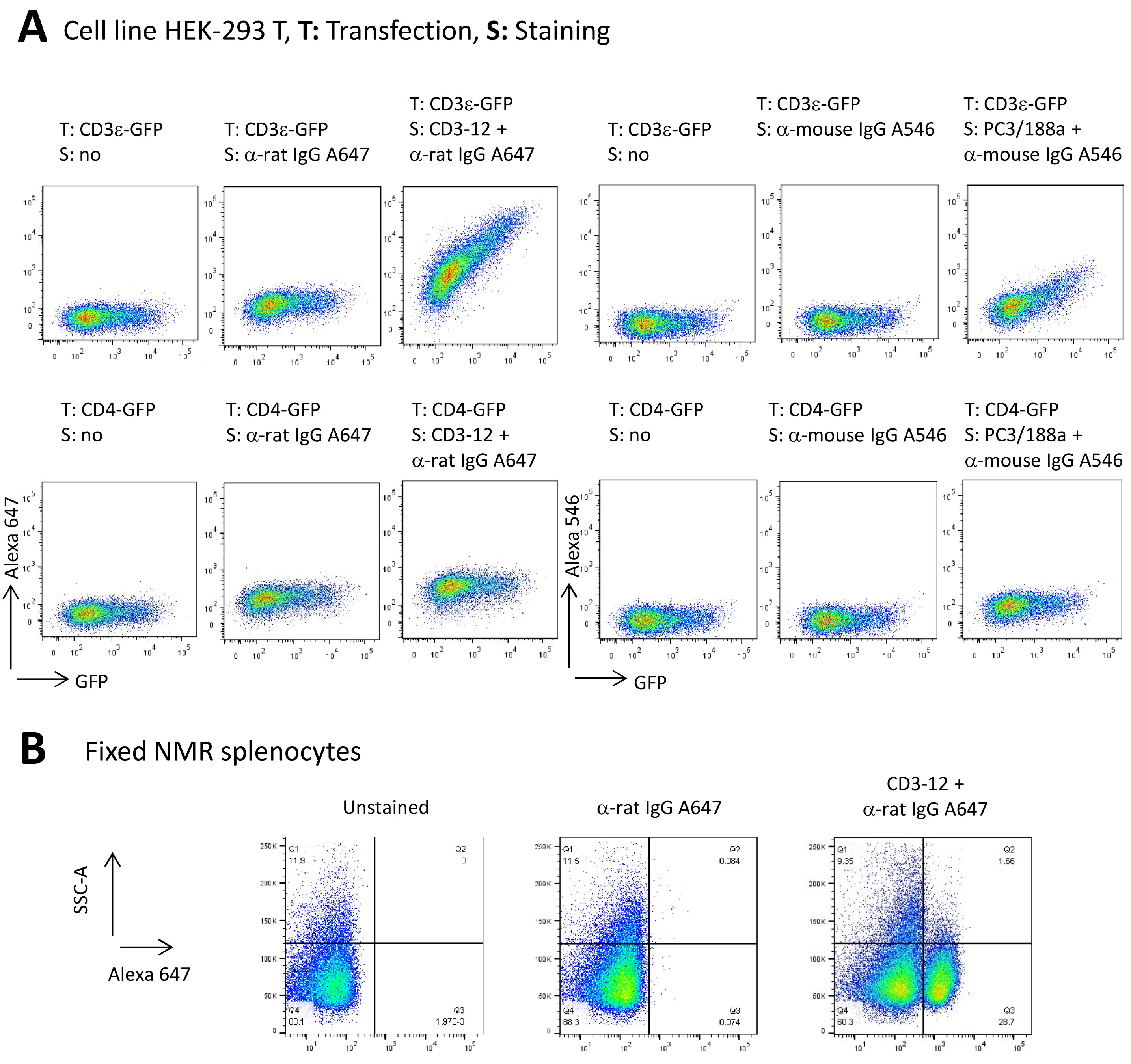

Supporting Information Figure S2. Antibodies recognising the intracellular domain of NMR CD3ε
A. HEK 293T human cells were transfected with vectors encoding NMR CD3ε and CD4 (used as negative control). Representative results from one of two experiments are shown.
B. Splenocytes isolated from NMR. **A** and **B.** Cells were fixed, permeabilised and stained with rat CD3-12 (**A** and **B**) and mouse PC3/188a (**A** only) antibodies recognising human CD3ε.
α-Rat IgG A647 and α-mouse IgG A546 secondary antibodies were used respectively. Cells were analysed after exclusion of debris, non-lysed erythrocytes, aggregated and dead cells (Supporting Information Figure S1). **B.** Representative results from one of two experiments with a total of two NMR samples are shown. (**A** and **B**) Gating strategy is shown in the Supporting Information Figure S4.

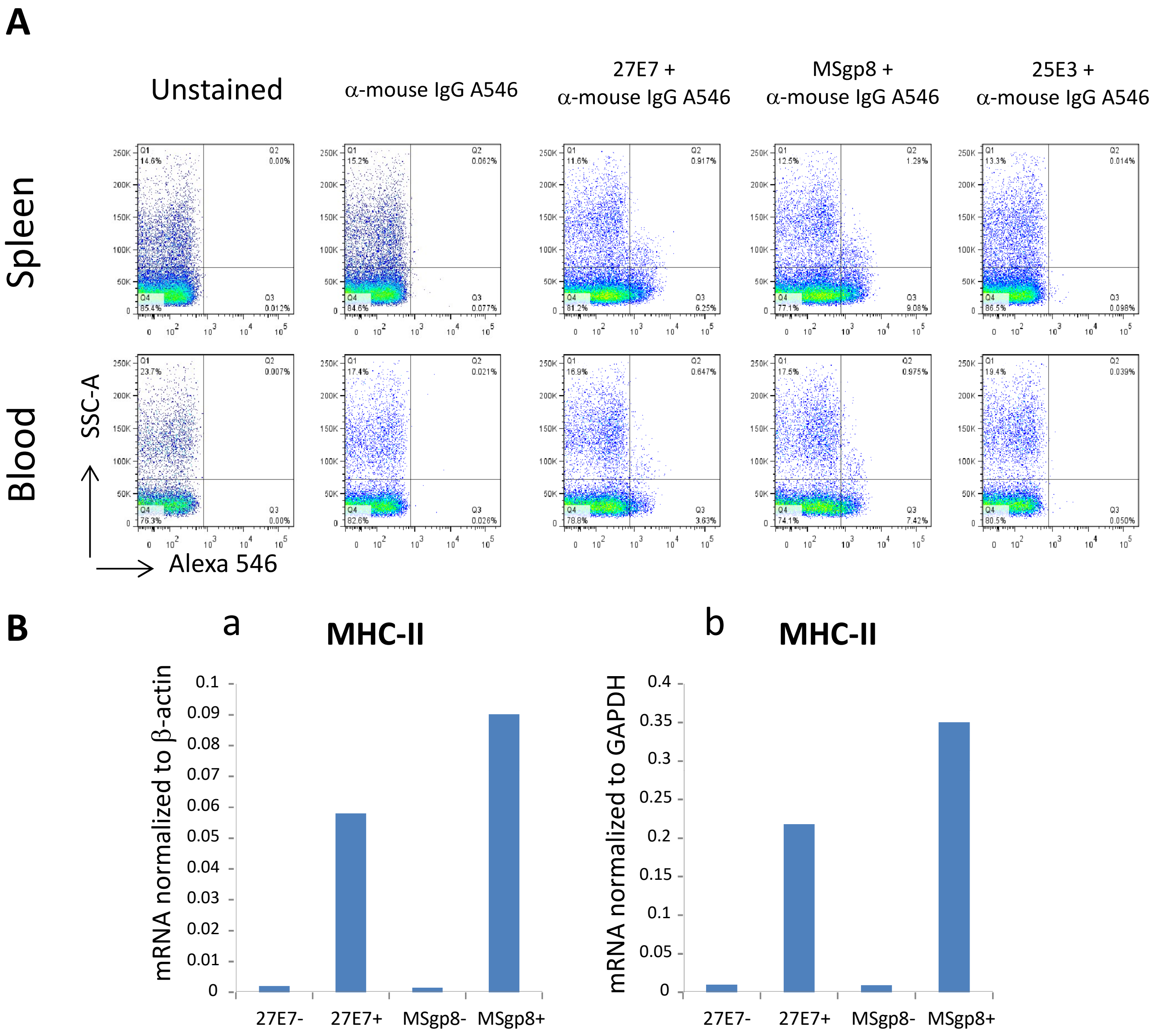

Supporting Information Figure S3. Antibodies recognising NMR MHC-II (example of Screening strategy II).

A. Cells were isolated from NMR blood and spleen, stained with antibodies, recognising Guinea pig MHC-II (clones 27E7, MSgp8 and 25E3 [used as negative control]) and secondary a-mouse IgG A546 antibodies. Cells were analysed by flow cytometry after exclusion of debris, non-lysed erythrocytes, aggregated and dead cells (gating strategy is shown in the Supporting Information Figure S4).

B. Cells, positive and negative for staining by antibodies against Guinea pig MHC-II clones 27E7 and MSgp8, were isolated by FACS from NMR bone marrow and analysed by Q-RT-PCR for expression of mRNA encoding NMR MHC-II antigen. PCR was set up in triplicates.

NMR β-actin (a) and GAPDH (b) were used as housekeeping genes for data normalisation.

(**A** and **B**) Representative results from one of two experiments with a total of two NMR samples are shown.

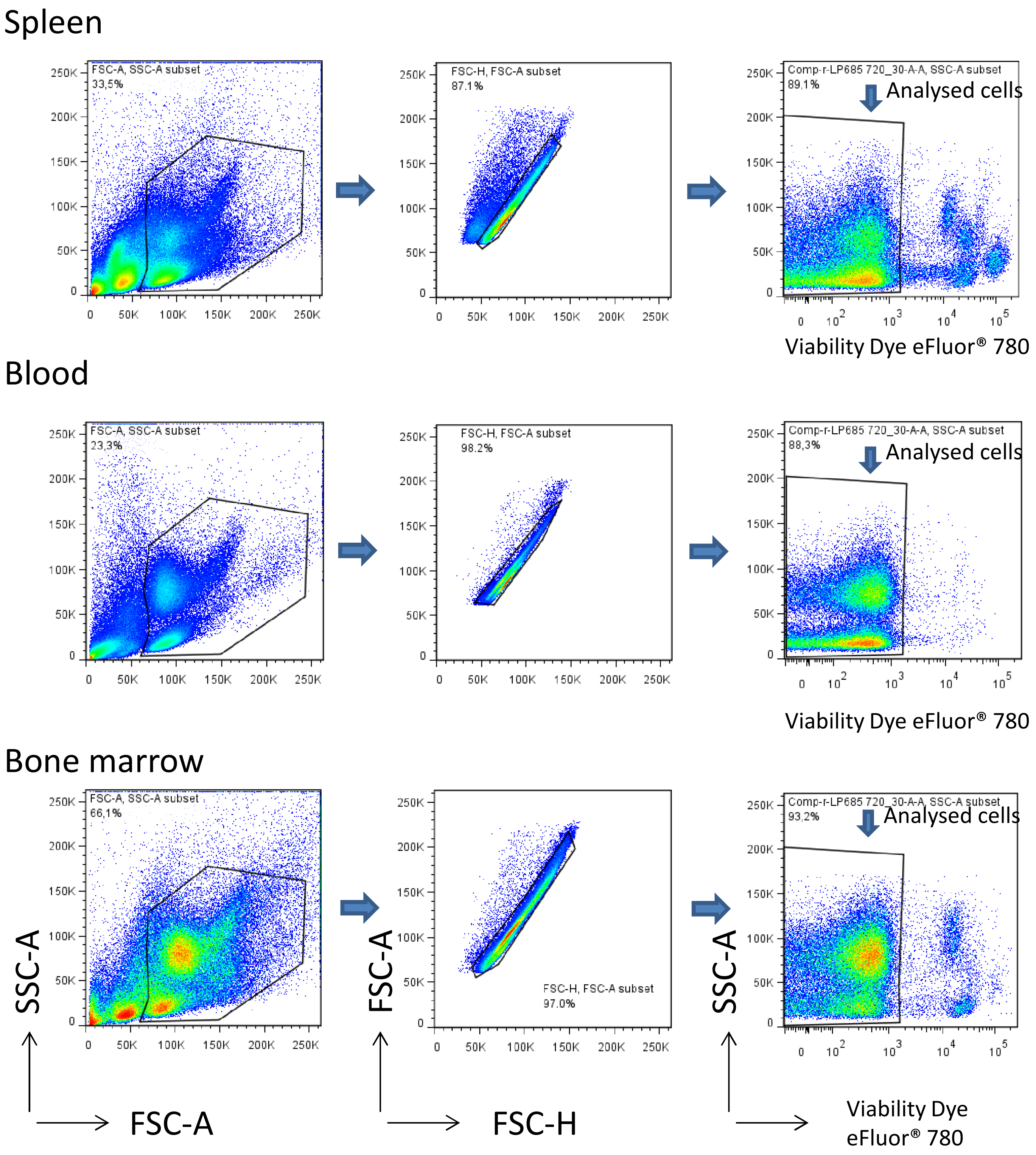

Supporting Information Figure S4. Gating strategy for conventional flow cytometry.

Cells were isolated from NMR spleen, blood and bone marrow.
Viability Dye eFluor® 780 was used for exclusion of dead cells.
Representative results from one of five independent experiments with a total of six NMR samples are shown.

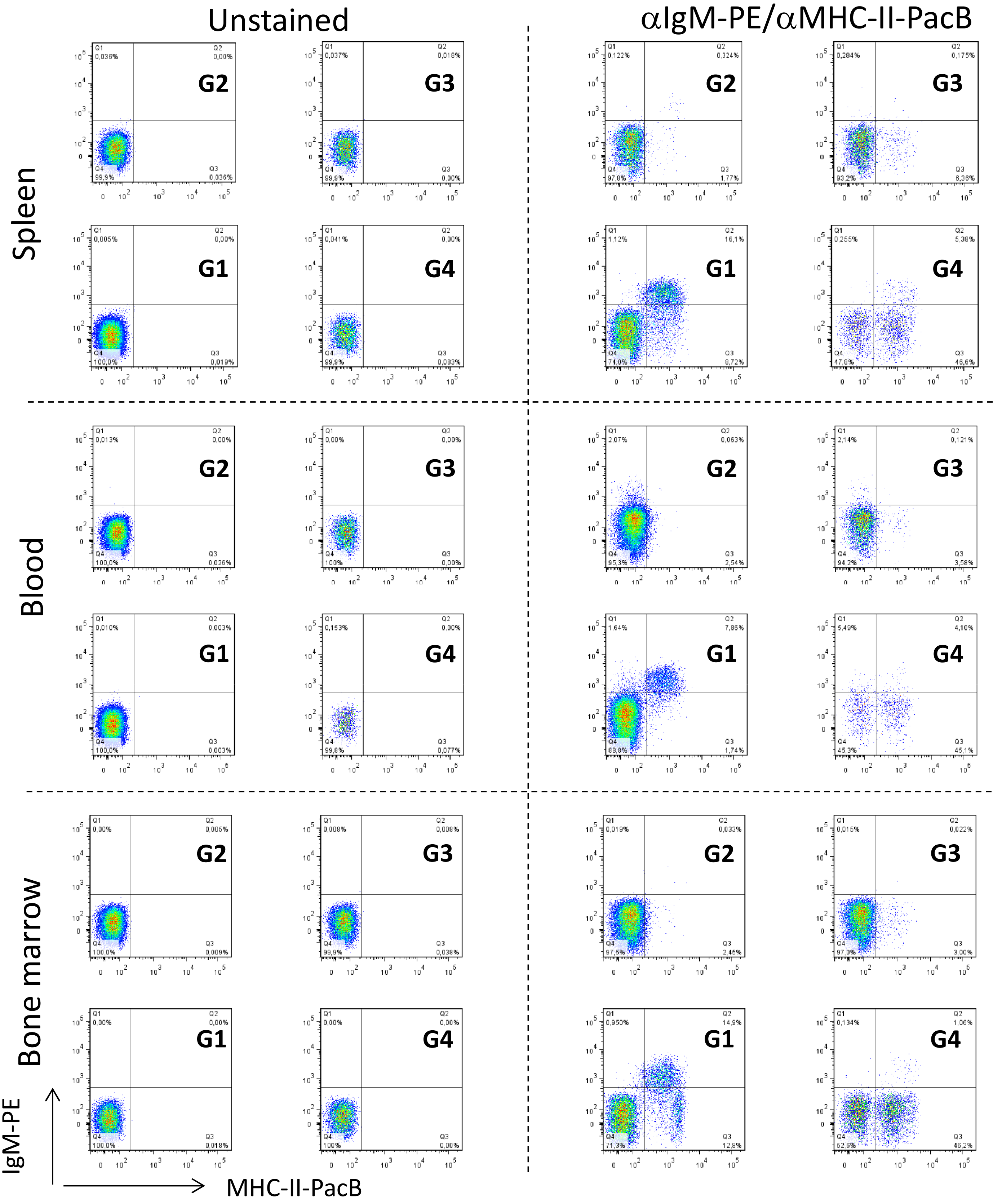

Supporting Information Figure S5. Identification of putative B cells (complete version of Figure 1C)
Cells were isolated from NMR spleen, blood and bone marrow and stained with antibodies recognising Guinea pig IgM (clone 31D2, conjugated with PE) and MHC-II (clone MSgp8, conjugated with PacB) (Table 1). G1 - G4 sub-population (Figure 1A) were analysed separately after exclusion of debris, non-lysed erythrocytes, aggregated and dead cells (gating strategy is shown in the Supporting Information Figure S4). Representative results from one of three experiments with a total of three NMR samples are shown.

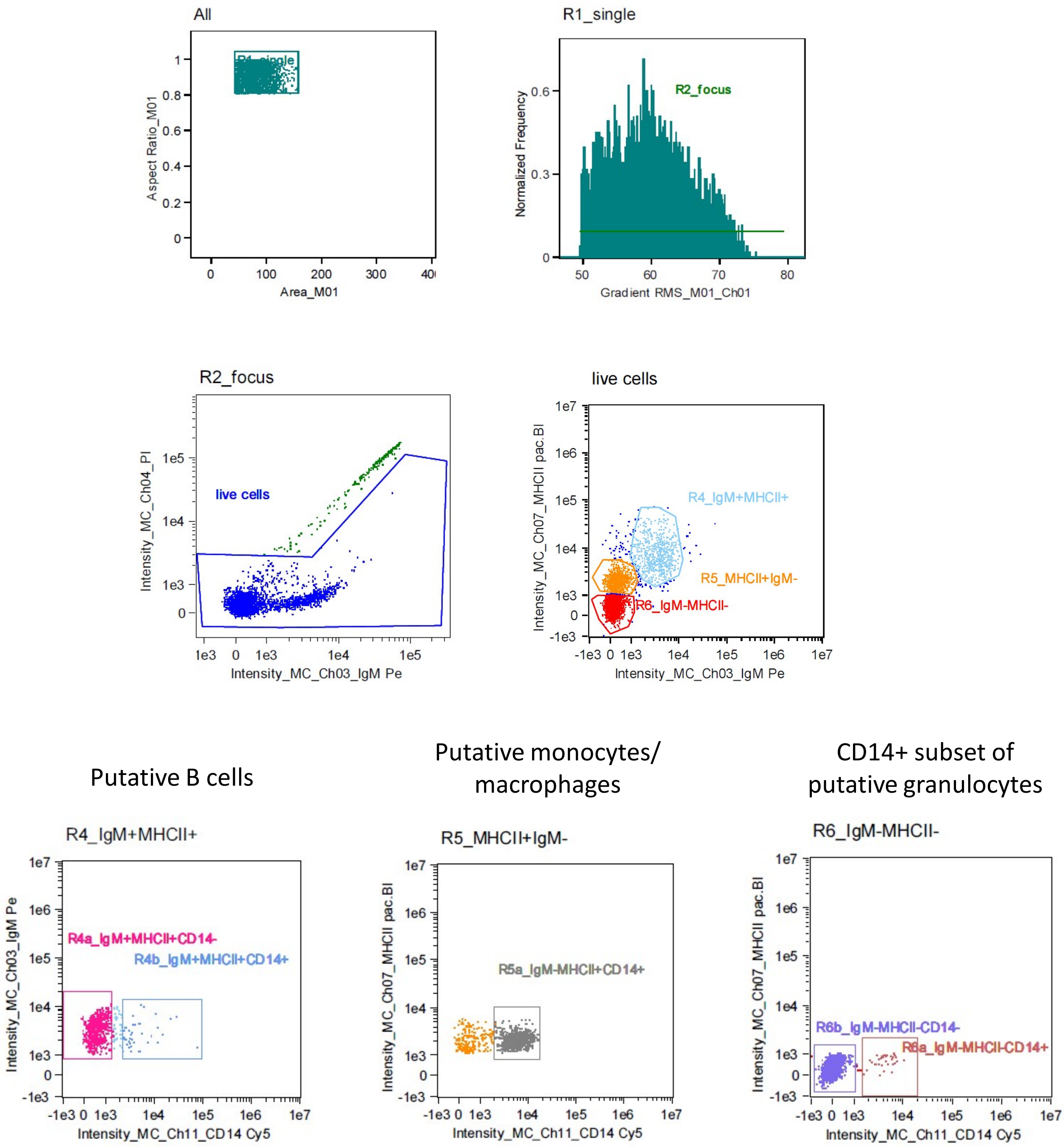

Supporting Information Figure S6. Gating strategy for imaging flow cytometry. Cells were isolated from NMR spleen, blood and bone marrow and stained with antibodies recognising Guinea pig MHC-II (clone MSgp8, conjugated with PacB) and IgM (clone 31D2, conjugated with PE) and human CD14 (clone TM1, conjugated with Cy5) (Table 1). Propidium iodide was used for exclusion of dead cells. A blood sample is shown as an example. Results from one of two experiments with a total of two NMR samples are shown.

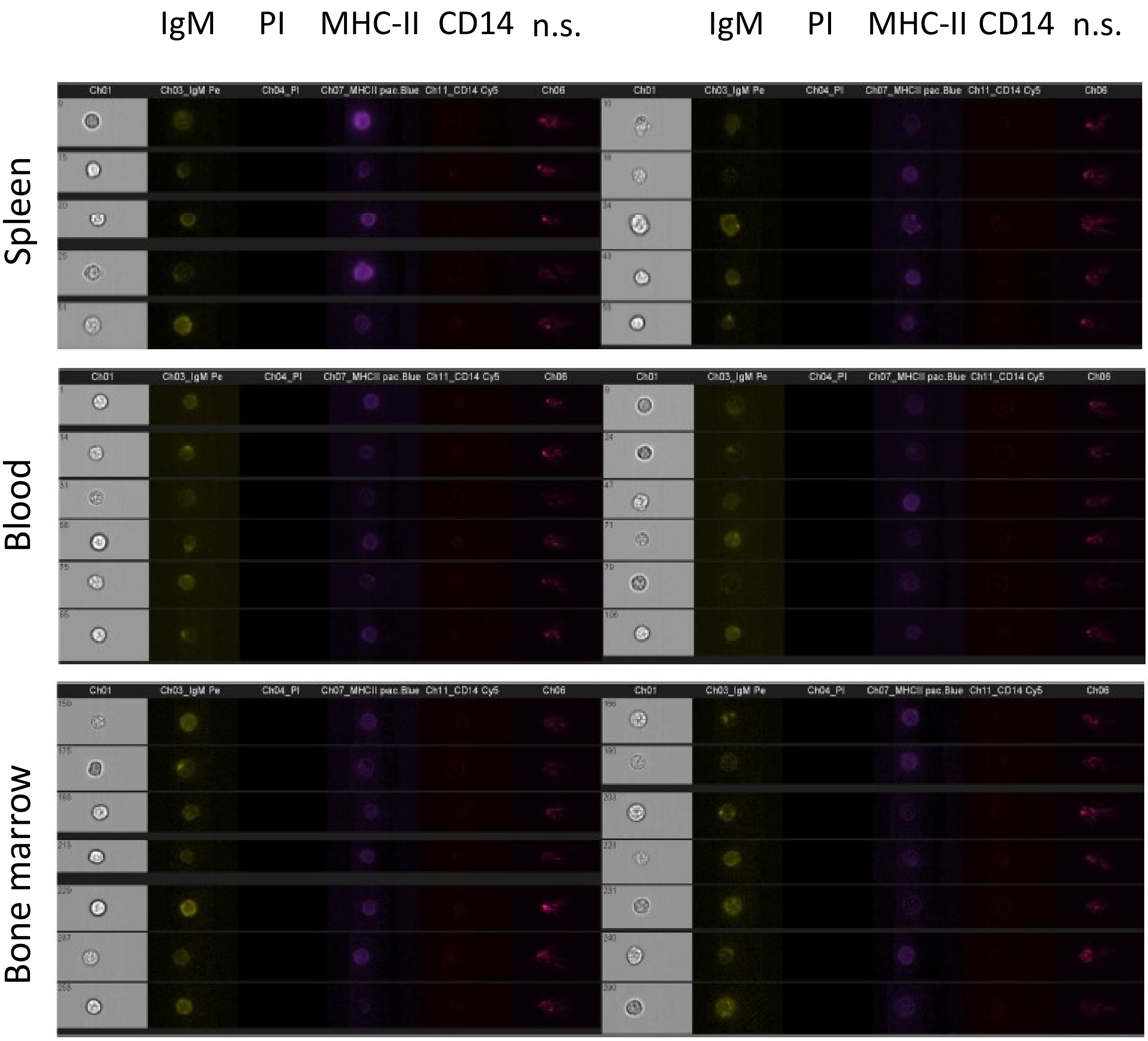

Supporting Information Figure S7A. Imaging flow cytometry analysis of NMR immune cells. Putative B cells.

Cells were isolated from NMR spleen, blood and bone marrow and stained with antibodies recognising Guinea pig MHC-II (clone MSgp8, conjugated with PacB) and IgM (clone 31D2, conjugated with PE) and human CD14 (clone TM1, conjugated with Cy5) (Table 1). Images of IgM$^+$MHC-II$^+$CD14$^-$ cells are shown after exclusion of debris, non-lysed erythrocytes, aggregated and dead cells (see the Supporting Information Figure S6 for gating strategy). Representative results from one of two experiments with a total of two NMR samples. n.s. – non-specific autofluorescence.

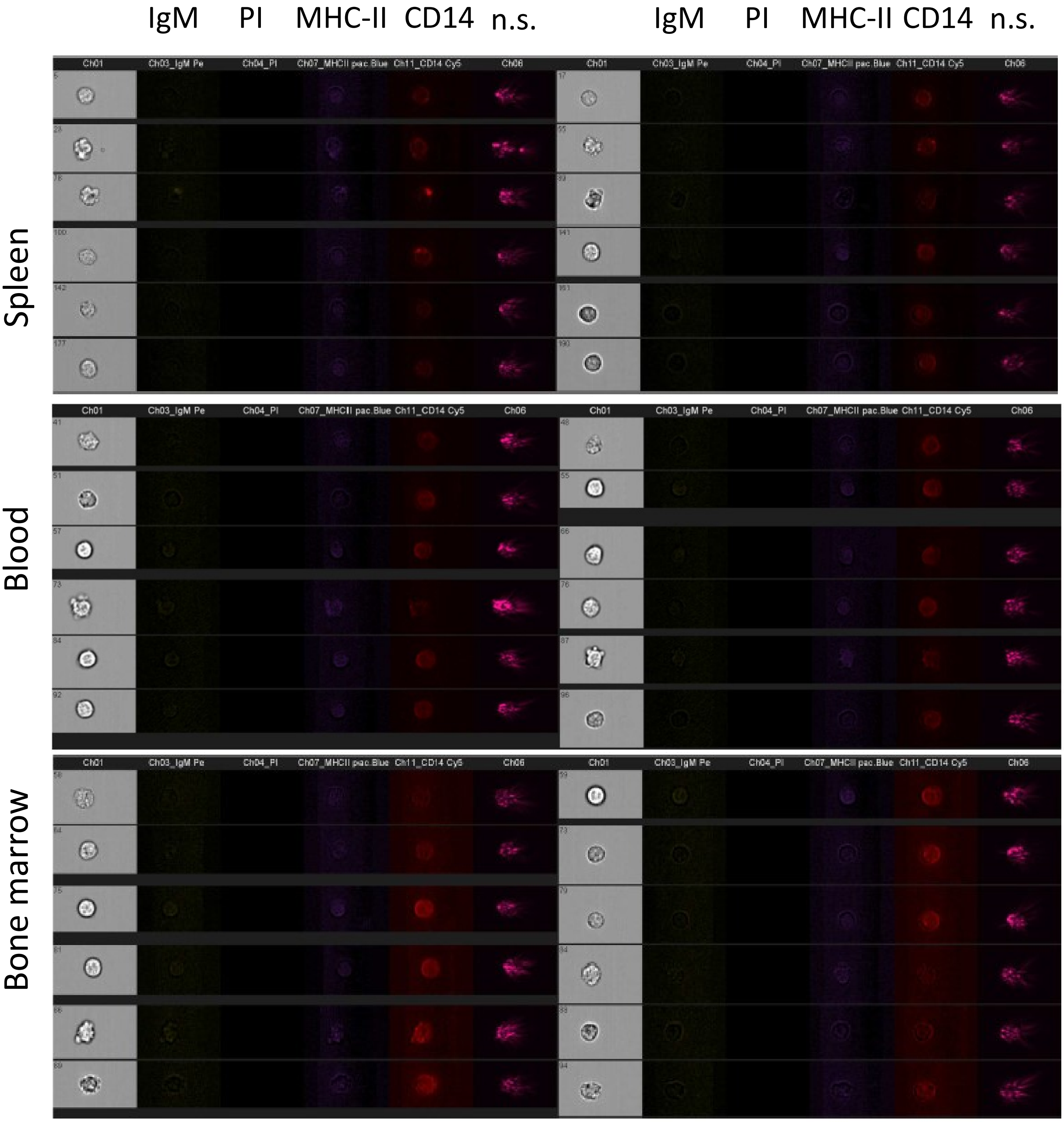

Supporting Information Figure S7B. Imaging flow cytometry analysis of NMR immune cells. Putative monocytes/macrophages.

Cells were isolated from NMR spleen, blood and bone marrow and stained with antibodies recognising Guinea pig MHC-II (clone MSgp8, conjugated with PacB) and IgM (clone 31D2, conjugated with PE) and human CD14 (clone TM1, conjugated with Cy5) (Table 1). Images of $IgM^{-}MHC\text{-}II^{+}CD14^{+}$cells are shown after exclusion of debris, non-lysed erythrocytes, aggregated and dead cells (see the Supporting Information Figure S6 for gating strategy). Representative results from one of two experiments with a total of two NMR samples. n.s. – non-specific autofluorescence.

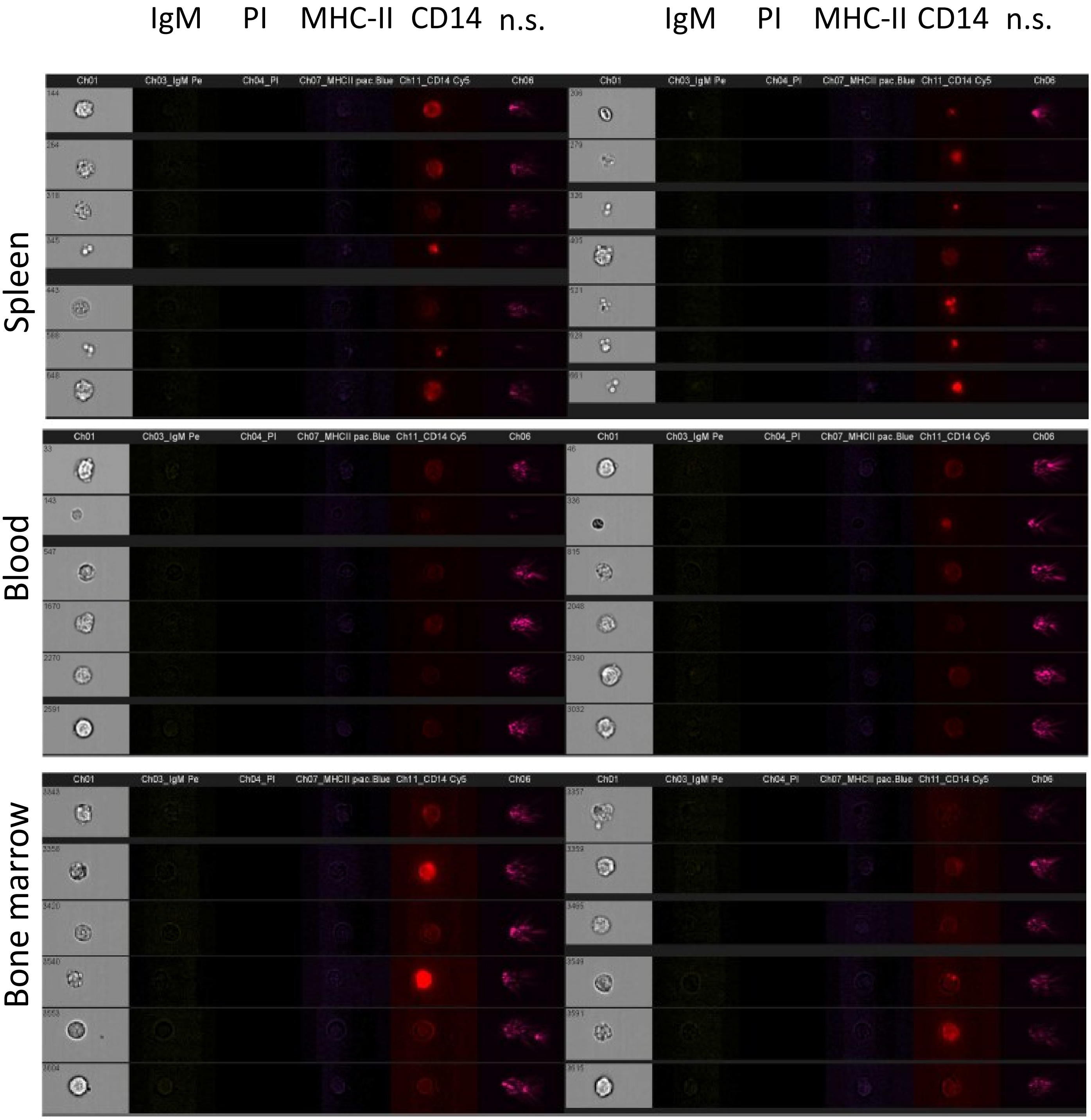

Supporting Information Figure S7C. Imaging flow cytometry analysis of NMR immune cells. $CD14^+$ subset of putative granulocytes.

Cells were isolated from NMR spleen, blood and bone marrow and stained with antibodies recognising Guinea pig MHC-II (clone MSgp8, conjugated with PacB) and IgM (clone 31D2, conjugated with PE) and human CD14 (clone TM1, conjugated with Cy5) (Table 1). Images of $IgM^-MHC\text{-}II^-CD14^+$cells are shown after exclusion of debris, non-lysed erythrocytes, aggregated and dead cells (see the Supporting Information Figure S6 for gating strategy). Representative results from one of two experiments with a total of two NMR samples. n.s. – non-specific autofluorescence.

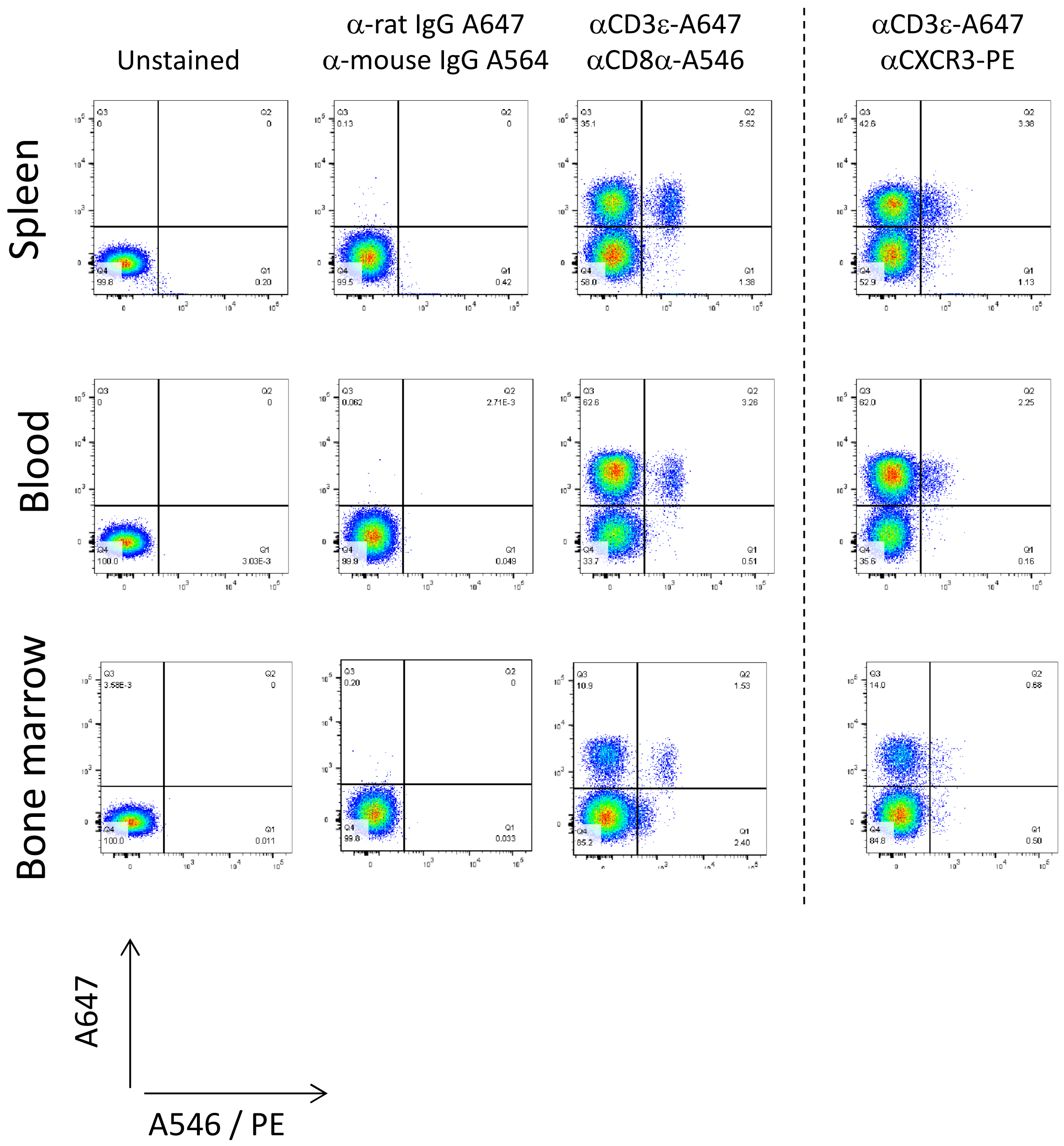

Supporting Information Figure S8. Identification of putative T cells (complete version of Figure 1E). Cells were isolated from NMR spleen, blood and bone marrow, pre-stained with antibodies, recognising Guinea pig CD8α (clone CT6, used with α-mouse IgG A546 secondary antibody) or mouse CXCR3 (clone CXCR3-173, conjugated with PE), fixed and stained with anti-CD3ε antibodies (clone CD3-12, used with α-rat IgG A647 secondary antibody). **G1** (FSC-A-low/SSC-A-low) sub-population (Figure 1A), representing lymphocytes, was analysed after exclusion of debris, non-lysed erythrocytes, aggregated and dead cells (gating strategy is shown in the Supporting Information Figure S4). Representative results from one of three experiments with a total of three NMR samples are shown.

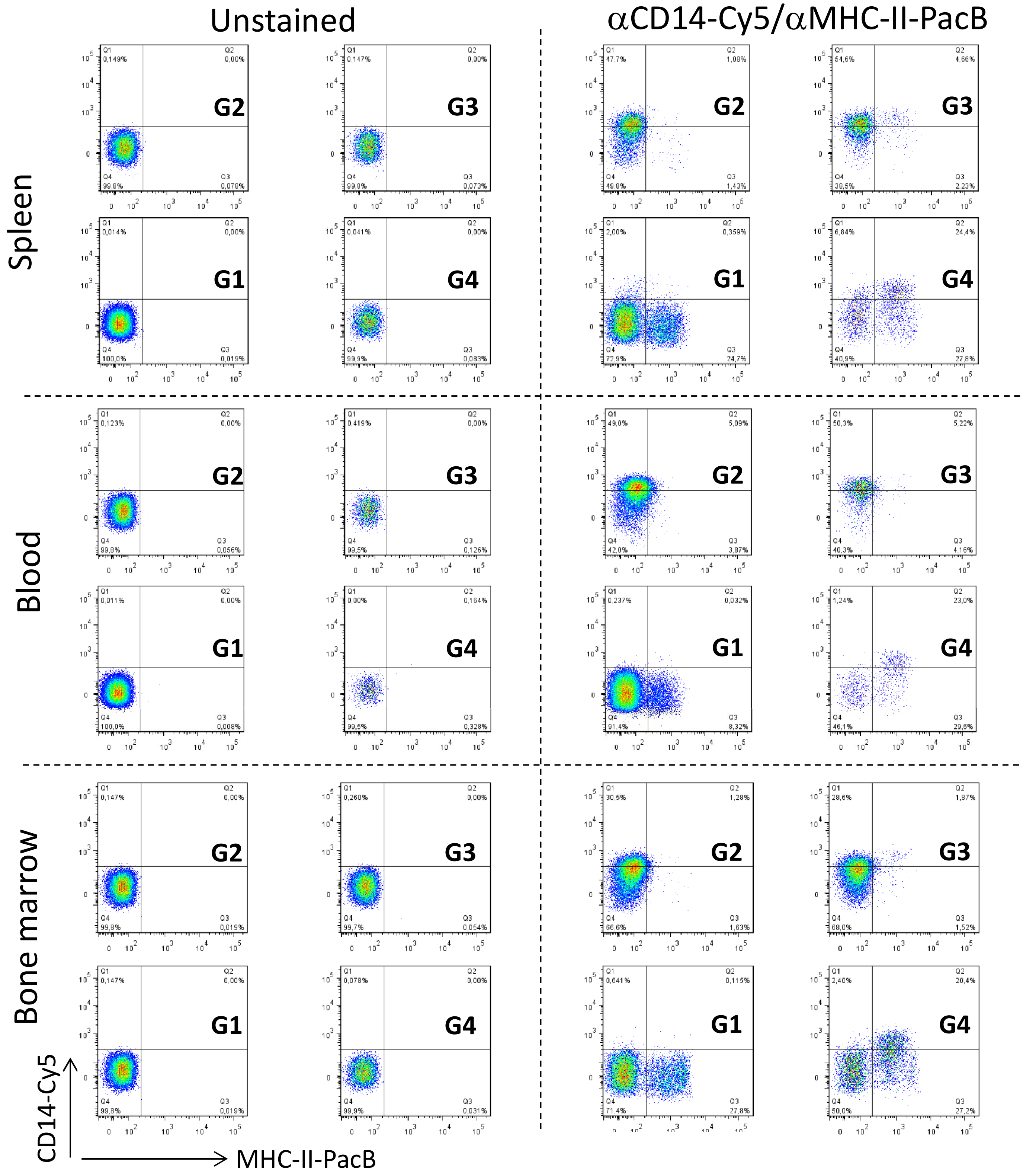

Supporting Information Figure S9. Identification of putative monocytes/macrophages (complete version of Figure 1F).

Cells were isolated from NMR spleen, blood and bone marrow, stained with antibodies recognising Guinea pig MHC-II (clone MSgp8, conjugated with PacB) and human CD14 (clone TM1, conjugated with Cy5) (Table 1) and analysed by conventional flow cytometry. G1- G4 sub-population (Figure 1A) were analysed separately after exclusion of debris, non-lysed erythrocytes, aggregated and dead cells (gating strategy is shown in the Supporting Information Figure S4). Representative results from one of three experiments with a total of three NMR samples are shown.

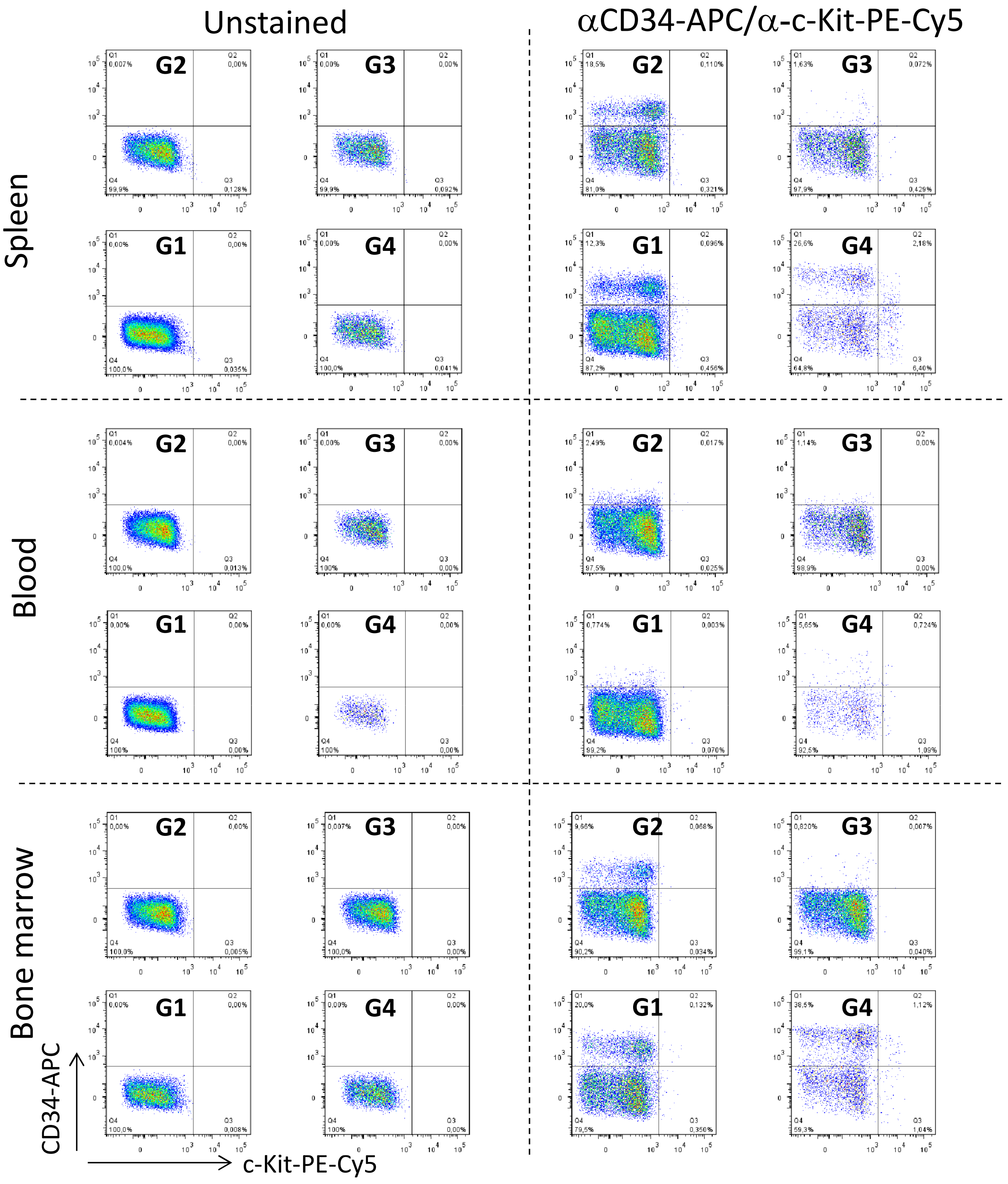

Supporting Information Figure S10A. Analysis of putative haematological precursors (complete version of Figure 1G).

Cells were isolated from NMR spleen, blood and bone marrow and stained with antibodies recognising human CD34 (clone AC136, conjugated with APC) and mouse c-Kit/CD117 (clone ACK4, conjugated with PE-Cy5) (Table 1). G1 - G4 sub-populations (Figure 1A) were analysed separately after exclusion of debris, non-lysed erythrocytes, aggregated and dead cells (gating strategy is shown in the Supporting Information Figure S4). Representative results from one of three experiments with a total of three NMR samples are shown.

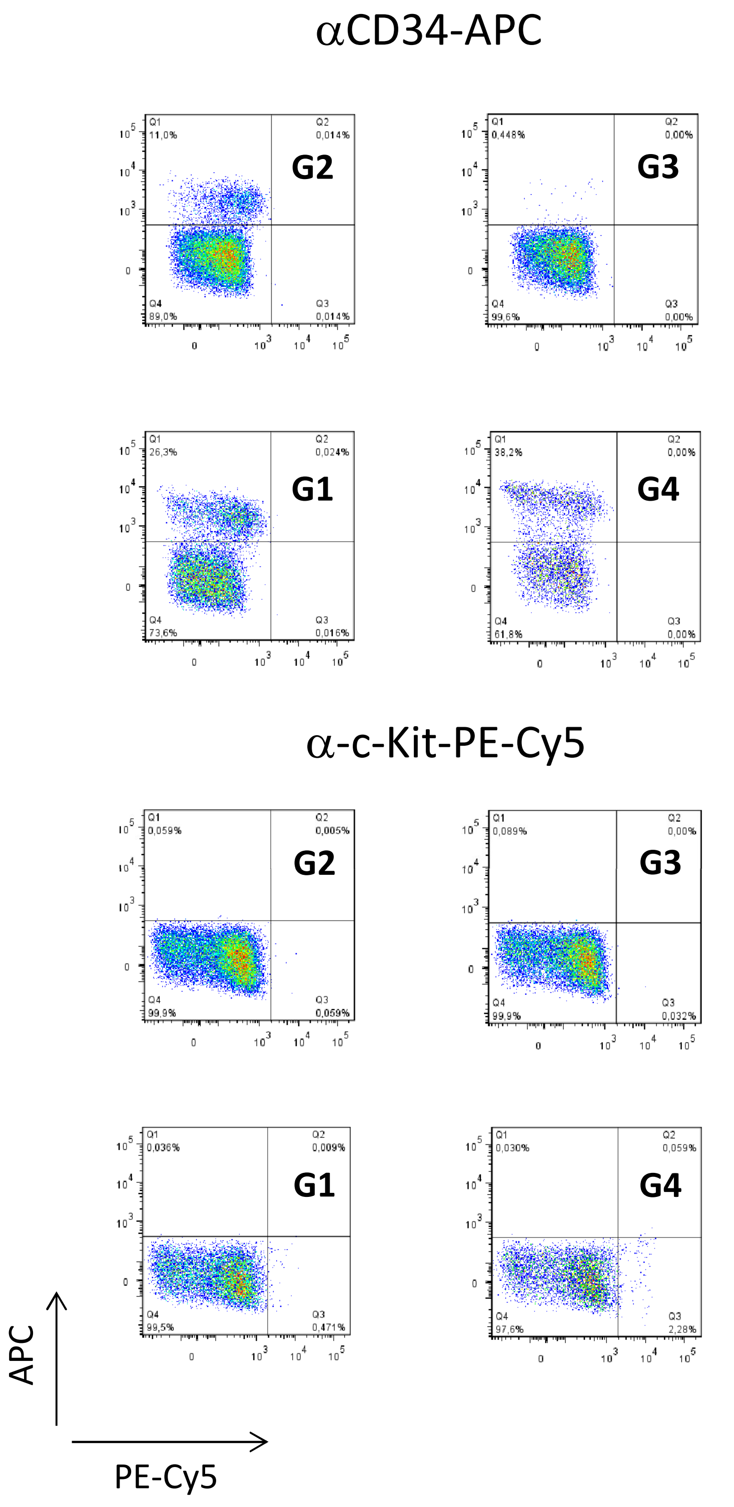

Supporting Information Figure S10B. Analysis of putative haematological precursors (individual stainings – control of channels compensation).
Cells were isolated from NMR bone marrow and stained with antibodies recognising human CD34 (clone AC136, conjugated with APC) or mouse c-Kit/CD117 (clone ACK4, conjugated with PE-Cy5) (Table 1). G1 - G4 sub-populations (Figure 1A) were analysed separately after exclusion of debris, non-lysed erythrocytes, aggregated and dead cells (gating strategy is shown in the Supporting Information Figure S4). Representative results from one of three experiments with a total of three NMR samples are shown.

Supporting Information - Table S1. Homology of NMR immune cells markers with Guinea pig, human, mouse and rat antigens

				Homology of extracellular domain compared with NMR					
Cell type marker	Cell type	Accession Number (NMR)	**Extracellular domain length**	Guinea pig	Human	Mouse	Rat	GP antibody available	Cloned
CD1b3 (GP only)	T cells	N/A	283 aa	N/A	N/A	N/A	N/A	Yes	
CD1c	mDCs	XP_004861888.1	284 aa	72%	70%	44%	46%		
CD3ε	T lymphocytes	XP_012932021.1	93 aa	60%	47%	59%	60%		Yes
CD3δ	T lymphocytes	XP_004856756.1	84 aa	75%	71%	62%	62%		Yes
CD4	T helpers	XP_004869501.1	368 aa	64%	56%	49%	48%	Yes	Yes
CD8α	T supressors	XP_004844626.1	159 aa	66%	55%	38%	39%	Yes	Yes
CD11c/ITGAX	DCs, monocytes, macrophages, neutrophils	EHB14281.1	1088 aa	79%	72%	69%	69%		
CD14	Monocytes	XP_004841948.1	326 aa	82%	75%	64%	65%		Yes
CD15/SSEA-1	Granulocytes	XP_012925097.1	353 aa	92%	85%	77%	81%		
CD16a/FCGR3A	Granulocytes, NK and monocyte subsets	XP_004865200.1	192 aa	71%	61%	63%	61%		Yes
CD19	B lymphocytes	XP_004856086.1	272 aa	81%	60%	63%	62%		Yes
CD20/MS4A1	B lymphocytes	XP_004874316.1	47 aa (biggest extracellular domain)	81%	79%	72%	74%		Yes
CD25/IL2RA	Activated T cells, Tregs	XP_004854755.1	216 aa	72%	56%	50%	53%		Yes
CD28	T lymphocytes	XP_004851403.1	134 aa	93%	77%	74%	77%		
CD31/PECAM1	Monocytes, neutrophils, platelets and some types of endothelial and T-cells	XP_012923088.1	603 aa	80%	69%	65%	65%		Yes
CD34	Hematopoietic, progenitor and stem cells	XP_004862097.1	263 aa	67%	60%	53%	49%		Yes
CD36/SCARB3	Monocytes	XP_004839812.1	410 aa	85%	83%	85%	86%		Yes
CD44/HCAM	Activated cells	EHB03001.1	639 aa	80%	71%	66%	66%		
CD45/PTPRC	Leukocytes	EHB03783.1	547 aa	58%	46%	40%	40%		Yes
CD49f/ITGA6	Hematopoietic stem cells (HSC), platelets, epithelial cells	XP_012932416.1	998 aa	88%	93%	90%	89%		
CD56/NCAM1	NK cells	XP_004856655.1	720 aa	99%	94%	94%	94%		
CD62L/LECAM1	Naive cells	XP_004865288.1	294 aa	82%	82%	76%	76%		Yes
CD66b/CEACAM8	Neutrophils	XP_004873064.1	286 aa	63%	63%	57%	52%		
CD69/CLEC2C	Activated cells	XP_004869592.1	138 aa	71%	64%	60%	60%		Yes
CD90/Thy-1	T lymphocytes	XP_004856862.1	111 aa	91%	75%	67%	71%	Yes	Yes
CD117/c-Kit	Hematopoietic progenitor and stem cells	EHB08085.1, XP_012929387.1	500 aa	93%	83%	78%	80%		Yes
CD120b/TNFR2	T regs	XP_004863597.1	235 aa	74%	64%	54%	50%		
CD138/Syndecan1	Plasma cells	XP_004839100.1	220 aa	66%	61%	56%	56%		
CD314/NKG2D	NK cells	XP_021114357.1	144 aa	77%	73%	69%	69%		
IgM	B lymphocytes	EHB10927.1	> 460 aa	N/A	66%	64%	68%	Yes	
MHC-I	All cells	XP_012928310.1	284 aa	75%	77%	72%	73%	Yes	
MHC-II	Macrophages, B lymphocytes	XP_004846929.1	191 aa	85%	83%	78%	79%	Yes	
TCR	T cells	N/A	N/A	N/A	N/A	N/A	N/A		
				Guinea Pig	Human	Mouse	Rat		
			Average	**74.9%**	**70.0%**	**64.8%**	**65.2%**		
			Standard Deviation	17.4%	12.2%	13.5%	14.1%		
				Homology of full-lengh protein					
G-type receptors			**Full-lengh protein length**	Guinea Pig	Human	Mouse	Rat		
CCR5/CD195	Th1	XP_004835086.1	353 aa	77%	75%	72%	72%		
CCR6/CD196	Th17	XP_012932137.1	351 aa	88%	81%	76%	77%		
CCR7/CD197	Naive T cells	XP_004859488.1	378 aa	85%	86%	84%	82%		Yes
CRTH2/CD294	Th2	XP_004874493.1	382 aa	79%	76%	73%	71%		
CXCR3/CD183	Th1, cytotoxic CD8$^+$ cells	XP_004872712.1	365 aa	89%	85%	82%	83%		
				Guinea Pig	Human	Mouse	Rat		
			Average	**84.3%**	**82.3%**	**79.7%**	**78.7%**		
			Standard Deviation	5.0%	5.5%	5.9%	6.7%		

Supporting Information - Table S2. Primers

Primers for Q-RT-PCR

Primer name	Sequence
β-Actin F	GCGCTCTTTCAGCCTTCTTT
β-Actin R	TTGGCATAGAGGTCCTTGCG
GAPDH F	TGACCCGTTCATTGACCTCAACTAC
GAPDH R	AAGATGGAGATGGCCTTGCCG
IgM soluble F	CCCCTAACGTGACCGTGTTC
IgM soluble R	TGAAGCCGGACCTCATAAGC
IgM membr. F	CTCCCAACGTGACCGTGTTT
IgM membr. F	ACAGTCTTTCCATCCCGCAG
MHC-II F	CTTCCTGCCCTCTACTGACG
MHC-II R	GCTTCGCTCTTCAAACTCCC
c-Kit/CD117 F	TGTCTGTGTCCAAACCCAGC
c-Kit/CD117 R	CTGCGCCCACAGAACTAGAC
CXCR3 F	ACTGCTATGCCCGAATCCTG
CXCR3 R	TAGGTGATAGGGGGTCCAGC

Primers for cloning

Primer name	Sequence
CD3δ F	ACCAGATCTATGAGAACAGGGAGTAACTGCA
CD3δ R	ACTCTCGAGTCACTTGTCATCGTCATCCTTGTAGTCTTTGCTCCGGTGCCAGTTTTC
CD3ε F	ACCAGATCTATGCAGTCGAGCACTCTCTGGA
CD3ε R	ACTCTCGAGTCACTTGTCATCGTCATCCTTGTAGTCGACTCCTCTCTGATTTAGGCCG
CD4 F	ACCAGATCTATGAAGGCAGGACTATGTTCCA
CD4 R	GGTCACTTGTCATCGTCATCCTTGTAGTCAGTGATATTCCGAGTCTTCTGCA
CD8α F	ACCAGATCTATGGCCCCGCGGGTGACC
CD8α R	ACTCTCGAGTCACTTGTCATCATCATCCTTGTAGTCGACGAATTTCCCTGAAGGGC
CD14 F	ACCAGATCTATGGAACTCGCTCCCGGCT
CD14 R	ACTCAATTGTCACTTGTCATCGTCATCCTTGTAGTCAGTCCTGATCACAGTGATGGTGCT
CD16 F	ACTAGATCTCAGTATGTGGCATCTGCCG
CD16 R	GGTCACTTGTCATCGTCATCCTTGTAGTCTTTGTCCTGGTCCCGGCTC
CD19 F	ACCGGATCCATGCCACCTCCTCTCCTCCT
CD19 R	TCGCCCTTGCTCACCATAGTCACTTTGAAGAATCTCCTGG (fusion with GFP)
CD20 F	ACTAGATCTATGGACTACAAGGATGACGATGACAAGATGGCAGCACCTGGGAATTC
CD20 R	AATCTCGAGTTAAGGAGAGCTGTCATTCTCTGC
CD25 F	ATCAGATCTATGGAGCCACGCTTGCTGAC
CD25 R	ACTCTCGAGTCACTTGTCATCATCATCCTTGTAGTCGATGGCTCTTCTACTCTTCTTCCA
CD31 F	ATCAGATCTTCAGGATGCGGCCAGTGT
CD31 R	ACTCTCGAGTCACTTGTCATCATCATCCTTGTAGTCAATCAGACTAAGCCAATATGGGGT
CD34 F	ATCAGATCTATGCTGCTCCGCGGGATG
CD34 R	ACTCTCGAGTCACTTGTCATCATCATCCTTGTAGTCCAATTCGGTATCAGCCACATG
CD36 F	ATCAGATCTATGGGCTGCGACCGGAAC
CD36 R	ACTCTCGAGTCACTTGTCATCATCATCCTTGTAGTCCTTCACGTTCTGTGTTCTGCATG
CD45 F	ACCAGATCTATGACAATGTATTTGTGGTTTAAACTTTTG
CD45 R	ACTCTCGAGTCACTTGTCATCATCATCCTTGTAGTCACTGAACACACGTGGGATGC
CD62L F	TATGGTATTTTCATGGAAATGTCA
CD62L R	ACTCTCGAGTCACTTGTCATCATCATCCTTGTAGTCATATGGGTCATTCATACTTTGCTG
CD69 F	TATGGACTACAAGGATGACGATGACAAGATGAATTCTGAAGAATGTCCCA
CD69 R	ACTCTCGAGTCATCTGGAGGCTCTGCTGC
CD90 F	ATTAGATCTATGAACCCAGCCATCGGC
CD90 R	ATACTCGAGTCACAGGGAAACGAAATCTGT
C-Kit/CD117 F	ATCAGATCTATGAGAGGCGCTCGCGGC
C-KIT/CD117 R	ACTCTCGAGTCACTTGTCATCATCATCCTTGTAGTCGGGTTTCTGTAAATATTTGTAGGT
CCR7/CD197 F	ATCAGATCTATGGACCCGGGGAAACC
CCR7/CD197 R	ACTCTCGAGTCACTTGTCATCATCATCCTTGTAGTCGGGAGAGAAGGTGGTGGTAG

Supporting Information - Table S3. Screened antibodies

Antigen	Clone	Note	availability in DRFZ or RKI	Fluorophore
CD1b3	MSgp9	anti-guinea pig	RKI	no
CD1c	L161	anti-human		PE-Cy7
CD3ε	HIT3a	anti-human		APC
CD3ε	UCHT1	anti-human	DRFZ	PE
CD3ε	BW264/56	anti-human		PE
CD3ε	SK7	anti-human		PE
CD3ε	OCT3	anti-human	DRFZ	A647
CD3ε	145-2C11	anti-mouse	DRFZ	PE
CD3ε	KT3	anti-mouse	DRFZ	PE, A647
CD3ε	PC3/188a	C-term, intracellular	RKI	no
CD3ε	CD3-12	C-term, intracellular		no
CD3δ	RIV9	anti-human		no
CD4	CT7	anti-guinea pig		PE
CD4	H155	anti-guinea pig	RKI	no
CD4	RPA-T4	anti-human		APC
CD4	TT1	anti-human	DRFZ	Cy5
CD4	SK3	anti-human		PE-Cy5.5
CD4	VIT4	anti-human		APC
CD4	OKT4	anti-human		BV650
CD4	9H5A8	anti-human		no
CD4	BL4	anti-human		no
CD8α	CT6	anti-guinea pig		no
CD8α	B607	anti-guinea pig	RKI	no
CD8α	GN11/134D7	anti-human	DRFZ	A647
CD11c	B-Ly6	anti-human		V450
CD11c	N418	anti-human		FITC
CD14	M5E2	anti-human		APC
CD14	TM1	anti-human	DRFZ	Cy5
CD15	HI98	anti-human		PE-Cy7
CD16	3G8	anti-human		PE
CD19	H1B19	anti-human		APC
CD19	BU12	anti-human	DRFZ	Cy-5
CD19	1D3	anti-mouse		Cy-5
CD20	2H7	anti-human		APC
CD20	REA294	anti-mouse		PE
CD20	LT20	anti-human		PE
CD20	1F54	anti-human	DRFZ	A405
CD25	M-A251	anti-human		APC
CD25	3G10	anti-human		PE
CD25	pC61.5	anti-mouse		Cy5
CD25	2A3	anti-human		PE
CD25	4E3	anti-human		PE
CD28	CD28.2	anti-human		FITC
CD28	37.51	anti-mouse		Cy5
CD31	156.1	anti-human	DRFZ	A405
CD31	158-2B3	anti-human	DRFZ	no
CD31	WM59	anti-human		PE
CD31	390	anti-mouse		A405

Supporting Information - Table S3 (cont.). Screened antibodies

Antigen	Clone	Note	availability in DRFZ or RKI	Fluorophore
CD34	AC136	anti-human		APC
CD36	AC106	anti-human		VioBlue
CD44	IM7	anti-human		PacBlue, Cy5
CD45	5B1	anti-human		PE-Vio770
CD45 RO	UCHL1	anti-human		Cy5
CD45 RA	4G11	anti-human		Cy5
CD45	HI-30	anti-human		V500
CD45	H201	anti-guinea pig	RKI	no
CD45R/B220	RA3.6B2	anti-human		A647
CD49f/ITGA6	GoH3	anti-human		PE
CD56	AF12-7H3	anti-human		APC
CD56	B159	anti-human	RKI	no
CD56	REA196	anti-human		PE
CD62L	145/15	anti-human		FITC
CD62L	DREG56	anti-human		PE
CD62L	Mel14	anti-mouse		APC
CD66b	REA306	anti-human	DRFZ	PE-Cy7
CD69	FN50	anti-human	DRFZ	PE-Cy7
CD69	H1.2F3	anti-mouse		PE-Cy7
CD90	167	anti-guinea pig	RKI	no
CD90	T24/31	anti-human	DRFZ	PacBlue
CD90	H154	anti-guinea pig	RKI	no
CD117/c-Kit	104D2	anti-human		BV605
CD117/c-Kit	ACK4	anti-mouse	DFRZ	Cy5, PE-Cy5
CD117/c-Kit	ACK45	anti-mouse	DRFZ	no
CD117/c-Kit	YB5.B8	anti-human		APC
CD120b/TNFR2	hTNFR-M1	anti-human		APC
CD138	MI15	anti-human		FITC
CD183/CXCR3	CXCR3-173	anti-mouse		PE
CD195/CCR5	2D7/CCR5	anti-human		PE-Cy7
CD196/CCR6	G034E3	anti-human		PE-Cy7
CD197/CCR7	4B12	anti-mouse		PE
CD197/CCR7	REA547	anti-human		PE
CD197/CCR7	G0437	anti-human		PE-Cy7
CD294/CRTH2	BM16	anti-human		PE
CD314/NKG2D	FAB139P	anti-human		PE
IgM	31D2	ant-guinea pig	RKI	PE
IgM	B621	ant-guinea pig	RKI	no
Macrophages	342	ant-guinea pig	RKI	no
MHC-I	22c4	ant-guinea pig	RKI	no
MHC-I	MSgp4	ant-guinea pig	RKI	no
MHC-II	L243	anti-human		PE-Cy7
MHC-II	G46-6	anti-human		PE-Cy5
MHC-II	27E7	anti-guinea pig	RKI	no
MHC-II	25E3	anti-guinea pig	RKI	no
MHC-II	MSgp8	anti-guinea pig	RKI	PacBlue
TCRαβ	T10B9	anti-human		FITC
TCRαβ	H159	ant-guinea pig		no
